# Construction of a chimeric lysin Ply187N-V12C with extended lytic activity against staphylococci and streptococci

**DOI:** 10.1111/1751-7915.12166

**Published:** 2014-09-15

**Authors:** Qiuhua Dong, Jing Wang, Hang Yang, Cuihua Wei, Junping Yu, Yun Zhang, Yanling Huang, Xian-En Zhang, Hongping Wei

**Affiliations:** 1 Department of Biomedical Engineering, College of Life Science and Technology, Huazhong University of Science and TechnologyWuhan, 430074, China; 2 Center for Emerging Infectious Diseases, Key Laboratory of Special Pathogens and Biosafety, Wuhan Institute of Virology, Chinese Academy of SciencesWuhan, 430071, China; 3 National Laboratory of Biomacromolecules, Institute of Biophysics, Chinese Academy of ScienceBeijing, 100101, China

## Abstract

Developing chimeric lysins with a wide lytic spectrum would be important for treating some infections caused by multiple pathogenic bacteria. In the present work, a novel chimeric lysin (Ply187N-V12C) was constructed by fusing the catalytic domain (Ply187N) of the bacteriophage lysin Ply187 with the cell binding domain (146-314aa, V12C) of the lysin PlyV12. The results showed that the chimeric lysin Ply187N-V12C had not only lytic activity similar to Ply187N against staphylococcal strains but also extended its lytic activity to streptococci and enterococci, such as *Streptococcus dysgalactiae*, *Streptococcus agalactiae*, *Streptococcus pyogenes*, *Enterococcus faecium* and *Enterococcus faecalis*, which Ply187N could not lyse. Our work demonstrated that generating novel chimeric lysins with an extended lytic spectrum was feasible through fusing a catalytic domain with a cell-binding domain from lysins with lytic spectra across multiple genera.

## Introduction

There is an ever-growing concern over the globe spread of antibiotic resistance among human and animal pathogens (Rice, 2008). To combat the resistant bacteria, it is well recognized that novel antimicrobials are needed. Among new agents in development, phage lysins seem promising for Gram-positive bacteria because of their high *in vitro* and *in vivo* antimicrobial efficiency, low occurrence of resistance, and wide availability from bacteriophages (Nelson *et al*., 2012; Schmelcher *et al*., 2012a,b; Shen *et al*., 2012). In general, phage endolysins of Gram-positive bacteria display a two-domain modular structure, which comprises an N-terminal catalytic domain (CD) and a C-terminal cell wall binding domain (CBD) (Nelson *et al*., 2012; Schmelcher *et al*., 2012a,b; Shen *et al*., 2012). Utilizing this property, chimeric lysins with a catalytic domain and a bacterial cell binding domain from different native lysins have been constructed to generate novel lysins to control pathogenic bacteria in a variety of environments (Manoharadas *et al*., 2009; Idelevich *et al*., 2011; Pastagia *et al*., 2011; Schmelcher *et al*., 2012a,b; Mao *et al*., 2013; Yang *et al*., 2014).

Most lysins reported so far have a narrow host range similar to that of their phages rendering them generally either species or genus specific. Such specificity can be influenced by its CBD, which is responsible for attaching the enzyme to its specific substrate in the bacterial cell wall via non-covalent binding of carbohydrate ligands (Loessner *et al*., 2002). Although the specificity is generally considered to be an advantage of a lysin since it would be utilized to kill only the pathogenic bacteria with little/no effects on the commensal flora, it may become a limitation for treating multiple infections. Therefore, there are needs to develop lysins with a wide, or purposely designed, lytic spectrum against a range of pathogenic bacteria.

Several native lysins have been identified with broad lytic activity more than one genus (Deutsch *et al*., 2004; Yoong *et al*., 2004; Son *et al*., 2012; Gilmer *et al*., 2013). One of them is lysin PlyV12, which can lyse not only *Enterococcus* but also several streptococcal and staphylococcal strains (Yoong *et al*., 2004). Recently, another lysin PlySs2 showed lytic activity against staphylococci, streptococci and *Listeria* (Gilmer *et al*., 2013).

While it appears that a CBD is necessary for the lytic activity of some lysins, this is not always the case. A number of enzymes have shown increased lytic activity upon removal of the CBD. One of them is the CD (1-157aa, Ply187N) from the lysin Ply187, which showed much higher activity than the parent full-length Ply187 (Loessner *et al*., 1999). More interesting, when fusing Ply187N with a SH3b CBD (Mao *et al*., 2013) or a non-SH3b-like CBD of phiNM3 lysin (Yang *et al*., 2014), the chimeric lysins showed even higher lytic activity than Ply187N.

In the present work, a novel chimeric lysin (Ply187N-V12C) was constructed by fusing Ply187N with the CBD (146-314aa, V12C) of PlyV12 to test if the lytic specificity of Ply187N would be extended to other genera by adding a CBD from a lysin with broad lytic activity.

## Results

### Construction and expression of the recombinant proteins

**Figure 1:**
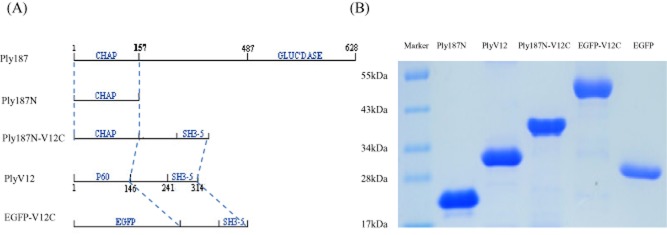
Schematic representation (A) and SDS-polyacrylamide gel electrophoresis analysis (B) of the recombinant proteins. All of the proteins contain a C-terminal His-tag used for affinity chromatography purification. The proteins migrate as expected for their predicted molecular weights: Ply187N, 18.9 kDa; PlyV12, 35.2 kDa; Ply187N-V12C, 37.2 kDa; EGFP-V12C, 47.9 kDa and EGFP, 29.2 kDa.

Using the standard genetic engineering methods, five recombinant proteins, i.e. Ply187N, PlyV12 and Ply187N-V12C, EGFP-V12C and EGFP, were expressed as shown in Fig. 1. It was found that all the proteins could be well expressed by *Escherichia coli*. After purification and dialysis, all the proteins displayed high purities (> 90%) in 12% SDS-PAGE gel (Fig. 1B).

### PlyV12 CBD can bind with enterococcal, streptococcal and staphylococcal strains

**Figure 2:**
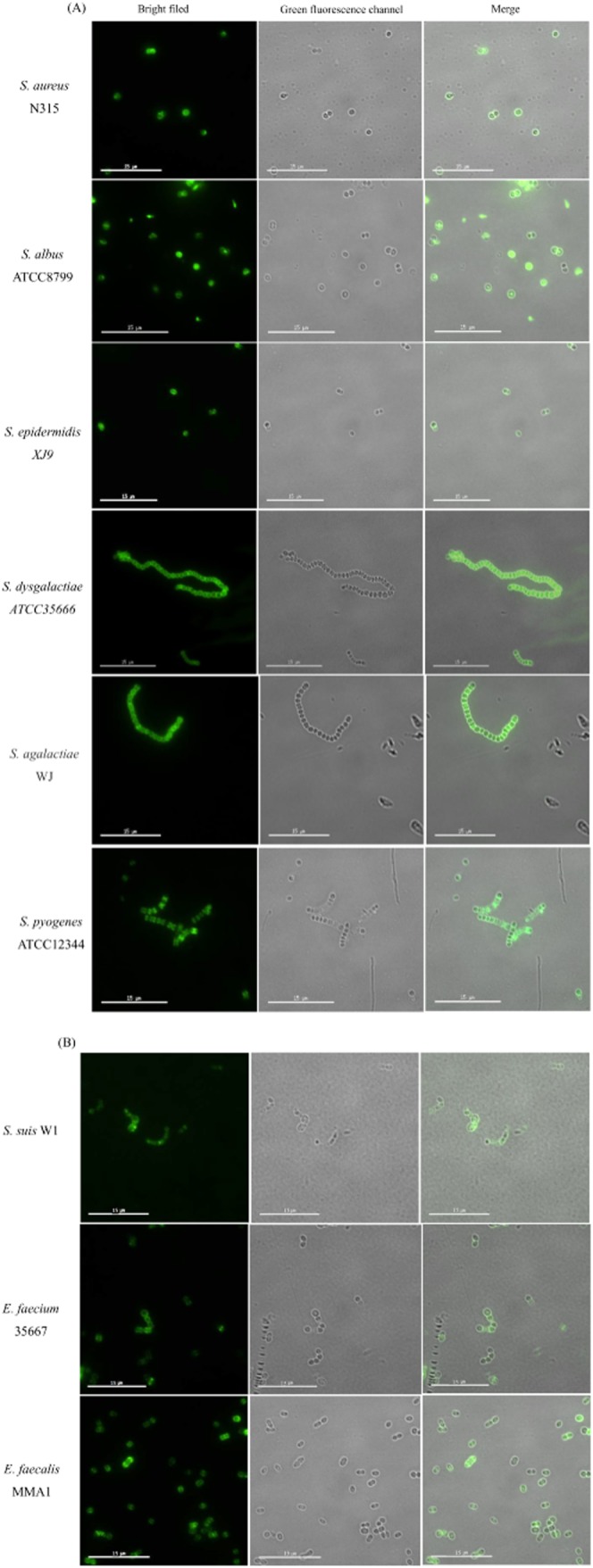
Fluorescence images of bacterial cells stained with EGFP-V12C. Bar size: 15 μm. All panels are viewed through a 60× magnification oil-immersion objective lens. The exposure time is set at 0.2 s.

Although PlyV12 was first reported as early as 2004, its CBD has not been studied. Through blasting the protein sequences in National Center for Biotechnology Information, the C-terminal of PlyV12 (146-314aa, V12C) was found homologous to the SH3-5 superfamily, which appears frequently in phage endolysins as CBD. As shown in Fig. 2, after incubation with the recombinant protein EGFP-V12C, all the cells of the staphylococcal, streptococcal and enterococcal strains tested were stained with green fluorescence. Under the same exposure time, no fluorescence could be seen after incubating the cells of these strains with EGFP respectively (data not shown). Furthermore, the cells of *Listeria monocytogenes, Bacillus cereus* and *E. coli* could not be stained by EGFP-V12C (data not shown). These results confirmed that the V12C fragment was the CBD of PlyV12, and the CBD of PlyV12 had a wide affinity to enterococci, streptococci and staphylococci, covering the host range of PlyV12 reported (Yoong *et al*., 2004). In the following experiments, only the staphylococcal, streptococcal and enterococcal strains in Table 1 were used to test the lytic activity of the three lysins.

**Table 1:** Bacterial strains used in this study and their susceptibility measured by the plate lysis assay to the recombinant lysins Ply187N, PlyV12 and Ply187N-V12C

	Strains	Source	Susceptibility^d^ to		
			Ply187N	PlyV12	Ply187N-V12C
*Staphylococcus*	*S. aureus* N315 (MRSA)	a	+	++	++
	*S. aureus* AM001 (MRSA)	a	+	++	++
	*S. aureus* AM002 (MRSA)	a	++	+++	+++
	*S. aureus* AM005 (MRSA)	a	+	++	+++
	*S. aureus* AM006 (MRSA)	a	+	++	+++
	*S. aureus* AM008 (MRSA)	a	++	++	+++
	*S. aureus* AM010 (MRSA)	a	+	+++	+++
	*S. aureus* AM014 (MRSA)	a	+	++	++
	*S. aureus* AM016 (MRSA)	a	+	++	++
	*S. aureus* AM027 (MRSA)	a	+	+++	+++
	*S. aureus* AM031 (MRSA)	a	++	++	+++
	*S. aureus* AM032 (MRSA)	a	++	++	+++
	*S. aureus* AM037 (MRSA)	a	+	++	+++
	*S. aureus* AM038 (MRSA)	a	+	+	+++
	*S. aureus* AM043 (MRSA)	a	+	++	+++
	*S. aureus* AM045 (MRSA)	a	+	++	+++
	*S. aureus* AM046 (MRSA)	a	+	++	+++
	*S. aureus* AM048 (MRSA)	a	+	++	+++
	*S. aureus* AM054 (MRSA)	a	+	++	++
	*S. aureus* AM058 (MSSA)	a	++	++	+++
	*S. aureus* AM061 (MSSA)	a	+	++	++
	*S. aureus* AB9118 (MSSA)	CCTCC^b^	++	++	+++
	*S. aureus M1*	c	+	+++	+++
	*S. aureus 391*	c	+	+++	+++
	*S. aureus 2080*	c	+	+++	+++
	*S. albus* 8799	ATCC	+	−	++
	*S. epidermidis XJ9*	a	+	++	+++
*Streptococcus*	*S. dysgalactiae 35666*	ATCC	−	+++	+++
	*S. agalactiae* WJ	c	−	+++	++
	*S. suis* W1	a	−	−	−
	*S. pyogenes* 12344	ATCC	−	+++	+++
*Enterococcus*	*E. faecium* 35667	ATCC	−	+/−e	−e
	*E. faecalis* MMA1	a	−	+	−e
Others	*L. monocytogenes* 19115	ATCC	−	−	−
	*B. cereus* 33018R	ATCC	−	−	−
	*E. coli* TG1	Invitrogen	−	−	−

a Lab collection.

b CCTCC: China Center for Type Culture Collection.

c Strains isolated from milk produced by cow with mastitis.

d A clear zone could be observed after overnight incubation at the amount of 5 pmol (+++), 50 pmol (++), or 500 pmol (+). −, no clear zone could be observed at the highest amount (500 pmol) tested.

e No clear zone was observed by the plate lysis assay, but lytic activity was seen by the microplate assay (Fig. 4).

ATCC, American Typical Culture Center; MRSA, methicillin-resistant S. aureus.

### Lytic activity of Ply187N-V12C, Ply187N and PlyV12

The lytic spectra of the lysins were screened using the plate lysis assay. Because Ply187N was reported specific to *Staphylococcus aureus*, the lytic activity of the chimeric lysin Ply187N-V12C was tested first on a collection of 24 *S. aureus* strains including methicillin resistant *S. aureus* strains and three strains isolated from milk produced by cow with mastitis. As shown in Fig. S1 and Table 1, Ply187N-V12C maintained lytic activity against all the *S. aureus* strains tested, similar to that of Ply187N. The minimum inhibition concentration (MIC) assay also showed that Ply187N-V12C had activity same to Ply187N with a MIC value of about 2.0 μM against *S. aureus* N315. In contrast, the MIC of PlyV12 was about 1.0 μM against *S. aureus* N315.

**Figure 3:**
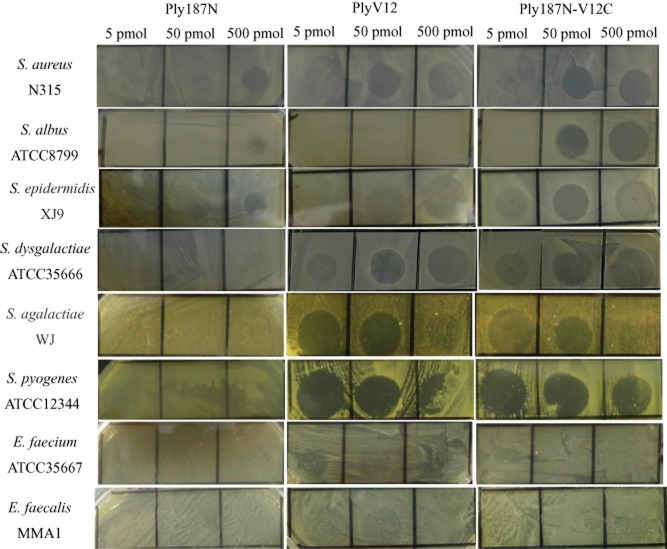
Inhibition zones formed on the plates after spotting with the three lysins at different concentrations.

**Figure 4:**
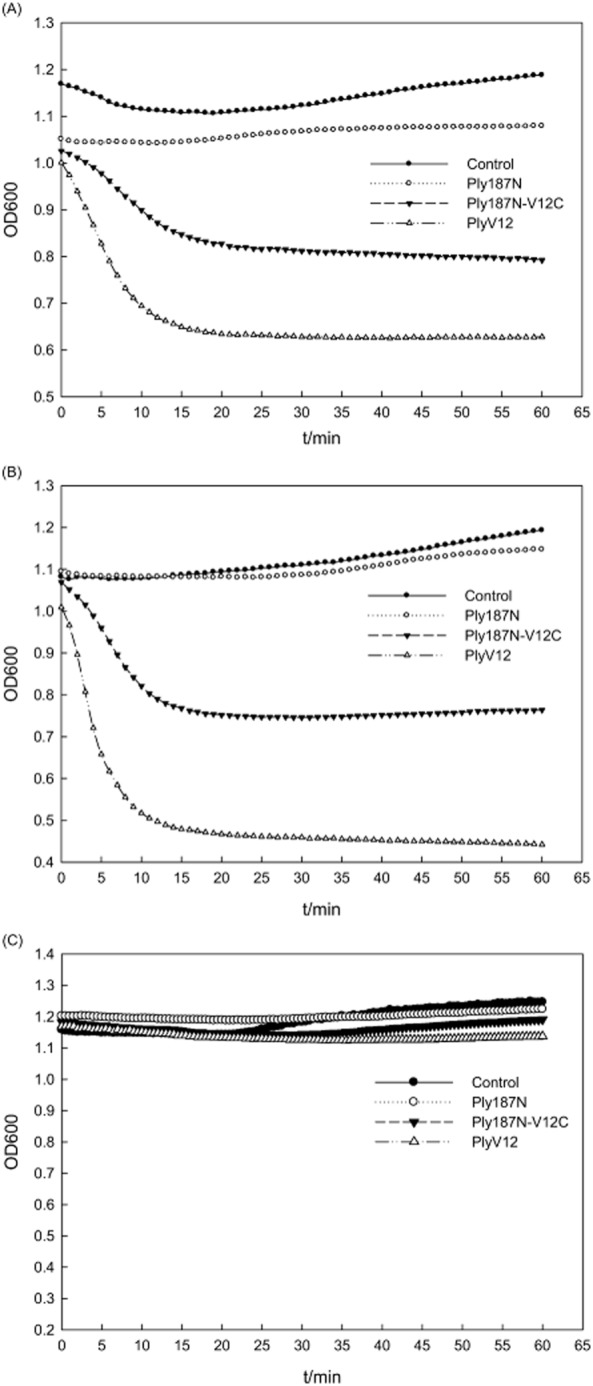
The lytic activity against *E. faecium* 35667 (A), *E. faecalis* MMA1 (B) and *S. suis* W1 (C) measured by the microplate assay. The concentration of the lysins is 2 μM in 5 mM Tris-HCl (pH 7.4).

Further tests were performed to study the lytic activity of the lysins on other bacteria from different genera listed in Table 1. It could be seen from Fig. 3 that Ply187N showed lytic activity just on the staphylococcal strains, while PlyV12 had activity on all the strains (faint zones on *Enterococcus faecium* 35667 and *Enterococcus faecalis* MMA1) except *Staphylococcus albus* 8799 by the plate lysis assay. In contrast, Ply187N-V12C showed lytic activity on all the strains except on *E. faecium* 35667 *and E. faecalis* MMA1 by the plate lysis assay. However, the microplate assay did show that Ply187N-V12C had lytic activity on the two enterococci strains, although less active than PlyV12 (Fig. 4A and B), which was not consistent with the plate lysis assay. Further MIC tests showed that Ply187N-V12C could inhibit the growth of *E. faecium* 35667 and *E. faecalis* MMA1 at about 5 μM, while PlyV12 at about 3 μM, which confirmed their lytic activity. However, no clear zones could be observed on the plates of *Streptococcus suis* W1 for all the three lysins at the highest amount of 500 pmol (data not shown). The microplate assay also confirmed that these three lysins didn't exhibit lytic activity against *S. suis* W1 (Fig. 4C). These results showed that the chimeric lysin Ply187N-V12C had an extended lytic activity not only on streptococci but also on some enterococcal strains compared with Ply187N, which could just work on staphylococci.

**Figure 5:**
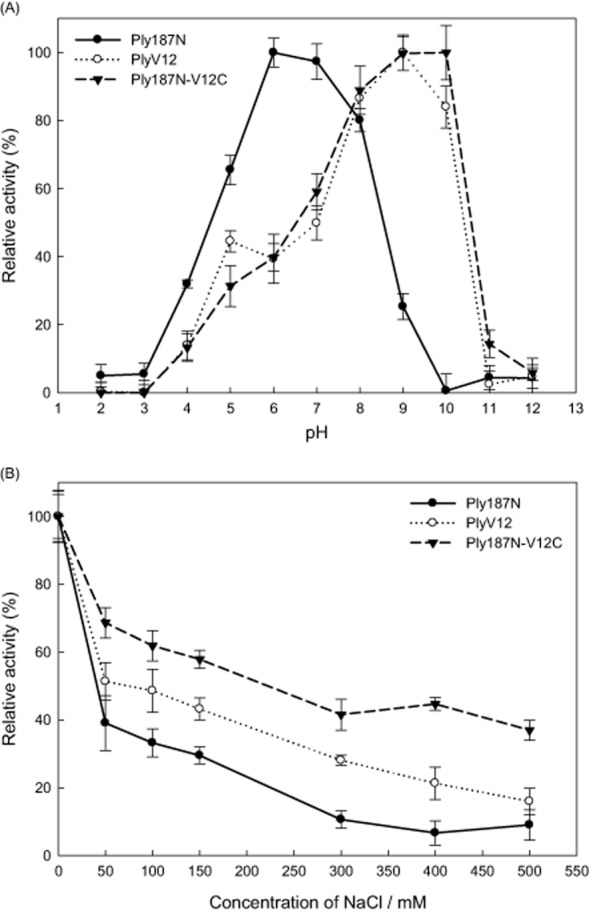
The relative activities of the three lysins in 20 mM boric acid and 20 mM phosphoric acid buffer at different pH (A) and in 5 mM Tris-HCl buffer (pH 7.4) with different ionic strength (B). The decrease in OD_600_ is monitored for 1 h after addition of lysis buffer or lysins to a 2 μM final concentration.

Using the microplate assay and *S. aureus* N315 as the test strain, the activity of Ply187N-V12C was found optimum around pH 9.0, which was higher than that of Ply187N (pH 6.0–pH 7.0) and similar to that of PlyV12 (Fig. 5A). As the concentration of sodium chloride (NaCl) in the lytic buffer increased, the activity of the lysins decreased gradually (Fig. 5B). Among the three lysins, Ply187N was found most sensitive to the ionic strength changes, while Ply187N-V12C was least sensitive to the ionic strength changes.

## Discussion

Developing novel chimeric lysins with a wide lytic spectrum would be important for some diseases caused by multiple bacterial infections. It has been widely recognized that novel chimeric lysins could be generated by swapping CDs and CBDs from different native lysins due to the modular structure of lysins. Because most native lysins are specific only to one certain genus, native lysins with lytic spectra across multiple genera would be unique sources for identifying CDs and CBDs suitable for generating novel chimeric lysins with a wide lytic spectrum.

In the current study, a novel chimeric lysin Ply187N-V12C with an extended lytic spectrum was successfully generated by fusing the CD from a staphylococcal lysin Ply187 with the CBD from a lysin with lytic spectra across multiple genera (PlyV12). Compared with their parental lysins, the activity of Ply187N-V12C was about the same to Ply187N and PlyV12 against staphylococcus, and slightly inferior to PlyV12 against enterococcus. Since *S. aureus*, *Staphylococcus dysgalatiae* and *Streptococcus agalactiae* are pathogenic bacteria normally found in cow mastitis (Barrett *et al*., 2005; Cha *et al*., 2014), this chimeric lysin might be useful for treating mastitis.

Because Ply187N itself showed no activity against non-staphylococci, it is clear that the binding domain V12C played a critical role on the extended activity of Ply187N-V12C against streptococci and enterococci. While in the previous two chimeric lysins consisting of Ply187N (Yang *et al*., 2014) (Mao *et al*., 2013), the lytic spectra were all limited to staphylococcal strains since the CBDs used are specific to Staphylococci. On the other hand, the optimum pH shift and the change of tolerance to the ionic strength indicated that the CBD V12C did not simply provide a binding to the bacterial cell wall but also had subtle impacts on the catalytic activity of Ply187N.

The extended activity of Ply187N-V12C against staphylococci, streptococci and enterococci also implies that the peptidoglycan structures in these three genera might share some similarity, which could be cleaved by the CHAP domain, i.e. Ply187N. CHAP is a common domain found in phage endolysins identified, which can either have a D-alanyl-glycyl endopeptidase (cleaves the D-Glu-L-Lys bond), or N-acetylmuramoyl-L-alanine amidase (cleaves the MurNAc-L-Ala bond) activity (Bateman and Rawlings, 2003). Notably, both bonds are conserved in streptococcal and staphylococcal peptidoglycan, which would be the reason why Ply187N-V12C could act on streptococci and staphylococci. However, as one could see from Figs 3 and 4C, both PlyV12 and Ply187N-V12C could not lyse *S. suis* W1, which the CBD could bind to (Fig. 2). These results indicated that *S. suis* W1 would share a common ligand for the CBD to recognize but might have some different peptidoglycan modifications which render Ply187N inactive. One thing worth to note is that no *S. suis* strains were tested in the previous study (Yoong *et al*., 2004), which makes it hard to tell whether *S. suis* strains would have some common bonds which PlyV12 could cleave. Further studies are needed to find the peptidoglycan difference between *S. suis* and other streptococcus to elucidate why Ply187N-V12C could not lyse the *S. suis* strain. Since there are only limited bond types in the peptidoglycan of bacterial cell walls (Loessner, 2005; Vollmer *et al*., 2008), it might be possible to use a few CDs to combine with one CBD with a wide-binding spectrum to generate chimeric lysins with predicted lytic spectra.

In conclusion, a novel chimeric lysin Ply187N-V12C with an extended lytic spectrum was successfully generated. Our work demonstrated that fusing a CD with a CBD from a lysin with a lytic spectrum across multiple genera was feasible for generating novel chimeric lysins with an extended lytic spectrum.

## Experimental procedures

### Bacterial strains and culture conditions

The bacterial strains used for this study are listed in Table 1. All the isolates were identified using an Omilog system (Biolog, USA). All the bacteria strains were cultured in Brain Heart Infusion (BHI, Becton Dickinson and Company, Sparks, MD, USA) at 37°C. *Escherichia coli* BL21 (DE3) was used for protein expression and grown in Luria-Bertani (LB) at 37°C. Kanamycin (50 μg ml^−1^) was used when necessary.

### Expression and purification of recombinant proteins

The DNA fragments encoding the lysin PlyV12 (Genbank accession No. AAT01859.1) and the N-terminal of endolysin Ply187 (Genbank accession No. CAA69022.1) were chemically synthesized by Songon Biotech (Shanghai, China). The gene fragment encoding the putative CBD (amino acid residues 146 to 314, V12C) was derived from the *plyV12* gene by polymerase chain reaction. Plasmids for expressing recombinant proteins Ply187N, PlyV12 and Ply187N-V12C were constructed by cloning the corresponding genes into the plasmid pET28a (Novagen, USA). Plasmids for expressing recombinant proteins enhanced green fluorescent protein (EGFP) and EGFP-V12C were purchased from Clontech Laboratories (Los Angeles, USA) or constructed by cloning the corresponding genes into the plasmid pET28a. The plasmids and primers used in this study are listed in Table S1. After confirmation by sequencing, the correct plasmids were transformed into *E. coli* BL21 (DE3) for expression of the recombinant proteins respectively. Deoxyribonucleic acid polymerase, restriction and modification enzymes were all purchased from New England Biolabs (Beijing, China) and used according to the manufacturer's instructions.

For producing the recombinant proteins, the *E. coli* BL21 (DE3) bacteria were incubated at 37°C to the mid-log phase first, then induced with 0.2 mM isopropyl β-D-thiogalactoside and finally incubated at 16°C overnight to allow expression. After collecting by centrifugation at 6000 *g* for 15 min, cells were lysed via sonication, and the His-tagged proteins were purified by HisTrap FF columns (GE Healthcare, USA) according to the supplier's instructions. Briefly, columns were washed by 20 mM imidazole after loading the proteins. Then, the target proteins were eluted with 250 mM imidazole. Finally, collected fractions were dialysed against 5 mM Tris-HCl (pH 7.4), filter sterilized and stored at 4°C until used.

### Cell wall binding assay and fluorescence microscopy of CBD of PlyV12

Cell wall binding activity of EGFP-V12C was observed by fluorescence microscopy (Loessner *et al*., 2002). Briefly, bacteria were harvested with centrifugation (12 000 *g*, 5 min), then washed and re-suspended with 1 × PBS (pH 7.4) for two times and finally re-suspended in 1 × PBS to a concentration of about 1 × 10^7^ CFU ml^−1^. After mixing 200 μl of the cell solution with 100 μl of 2 μM EGFP–V12C in 1 × PBS buffer, the mixture was incubated at 37°C for 30 min. As a control, the cells in another tube were mixed with EGFP and incubated at 37°C for 30 min. After the incubation, the cells were separated from the supernatants by centrifugation (12 000 *g*, 5 min), washed three times with PBST (1 × PBS with 0.5% tween-20) buffer and re-suspended in 1 × PBS before fluorescence microscopy. The fluorescence images were obtained using a Delta Vision Personal DV microscope (Applied Precision, USA) with a 60× magnification oil-immersion objective lens. The exposure time used was set at 0.2 s when capturing all the fluorescence images. Individual images were obtained using suitable filter settings, and colour channels were processed using the image processing software installed with the microscope. In the experiments, EGFP was incubated with the cells of the individual strains, respectively, as the negative control.

### Determining lytic activity of recombinant proteins

The lytic spectra of Ply187N, PlyV12 and Ply187N-V12C were tested using the plate lysis assay as described previously (Mao *et al*., 2013). Briefly, bacteria strains were grown to mid-log phase and spread on BHI agar plates as a lawn just before the test respectively. Then, 10 μl of the lysins with different concentrations (diluted in sterile 5 mM Tris-HCl buffer, pH 7.4) was spotted onto the plates respectively. The spotted plates were air-dried for 10 min in a laminar flow hood and incubated overnight at 37°C. Lytic activity was indicated by a clear zone formed on each plate.

The lytic activities of the proteins were also measured quantitatively using the microplate assays as previously described (Nelson *et al*., 2001), with some modifications. Briefly, the enterococcal strains and *S. suis* W1 were grown to an optical density of 0.2 to 0.3 at OD_600_, centrifuged, and re-suspended in 5 mM Tris-HCl (pH 7.4) to a final OD_600_ of 1.0. Then, 100 μl of the bacterial suspension were mixed with 100 μl of the purified proteins in 96-well plates (Perkin-Elmer, Waltham, MA, USA) to a final concentration of 2 μM respectively. The drop of OD_600_ was monitored at 30 s or 1 min intervals by a microplate reader (Synergy H1, BioTek, USA) for 60 min at 37°C.

Effects of ionic strength and pH on the lytic activities were also measured with *S. aureus* N315 using the microplate assay described above. In the ionic strength assay, concentrated NaCl solutions were diluted using 5 mM Tris-HCl buffer (pH 7.4) to prepare solutions with different NaCl concentrations. A universal buffer described before (Schmitz *et al*., 2011) was prepared by mixing equal aliquot of 20 mM boric acid and 20 mM phosphoric acid, followed by titration with sodium hydroxide from pH 2 to 12 for testing the pH effects on the lytic activities. After 60 min incubation, the lytic activity of the lysin under different conditions was calculated using the formula (OD600_0_-OD600_60_)/OD600_0_ respectively. Finally, the activity relative to the highest activity (100%) was drawn versus different NaCl concentrations or pH values to find the optimum conditions.

### Minimal inhibitory concentration

The minimum inhibitory concentrations of the three lysins against *S. aureus* N315 were measured in Mueller-Hinton culture as described before (Becker *et al*., 2009). Twofold serial dilutions of lysins from 5 μM to 0.031 μM were mixed with bacteria suspensions (about 1 × 10^5^ CFU ml^−1^ diluted from overnight bacterial culture). Minimum inhibition concentration was defined as the lowest concentration of the lysins producing inhibition of visible growth.

## Supporting Information

**Fig. S1.** Inhibition zone of *S. aureus* strains in the plate lysis assay. *S. aureus* M1, 391and 2080 are isolated from milk produced by cow with mastitis. **Table S1.** Plasmids and primers used in this study.
