# Supplementary material for: Construction of a chimeric lysin Ply187N-V12C with extended lytic activity against staphylococci and streptococci
Source: Microb Biotechnol. 2014 Sep 15;8(2):210–20. doi: 10.1111/1751-7915.12166 (PMC4353335; doi:10.1111/1751-7915.12166)
Supplement: Supplementary file 1 [file mbt20008-0210-sd1.docx]

**Construction of a Chimeric Lysin Ply187N-V12C with Extended Lytic Activity against Staphylococci and Streptococci**

Qiuhua Dong^1,2^, Jing Wang^2^, Hang Yang^2^, Cuihua Wei^2^, Junping Yu^2^, Yun Zhang^2^, Yanling Huang^2^, Xian-En Zhang^3*^, Hongping Wei^2**^,

1. Department of Biomedical Engineering, College of Life Science and Technology, Huazhong University of Science and Technology, Wuhan, 430074, China; 2. Center for New Emerging Infectious Diseases, Wuhan Institute of Virology, Chinese Academy of Sciences, Wuhan, 430072, China; 3. National Laboratory of Biomacromolecules, Institute of Biophysics, Chinese Academy of Science, Beijing 100101

**For correspondence. *E-mail: hpwei@wh.iov.cn; Tel/Fax: (+86) 27 51319676; or **E-mail: x.zhang@wh.iov.cn; Tel.: (+86) 27 87199115; Fax: (+86) 27 87199492.**

The **Supplemental material** contains the following content:

**Table S1. Plasmids and primers used in this study.**

**Figure S1. Inhibition zones of *S. aureus* strains in the plate lysis assay.**

**Table S1. Plasmids and primers used in thi study**

| **Plasmids** | **Vector** | **Cloning site** | **Relevant properties** | **Sources** |
| --- | --- | --- | --- | --- |
| pEGFP | pEGFP-C1 | *Nhe* I */ Bsr*G I | For expression of EGFP | Clontech |
| pEGFP-V12C | pET28a | *Nde* I */ Bam*H I  and *Eco*R I */ Xho* I | For expression of EGFP-V12C | This study |
| p187N | pET28a | *Nco* I */ Xho* I | For expression of Ply187N | Our Lab. |
| p187NV12C | pET28a | *Nco* I */ Bam*H I  and *Eco*R I */ Xho* I | For expression of Ply187N-V12C | This study |
| pV12 | pET28a | *Nco* I */ Xho* I | For expression of PlyV12 | This study |
| **Target gene** | **Primer** | **Sequence (5' to 3')** | | |
| *egfp* | egfp-f | AAAACATATGGCTACCGGTCGCCACCATGGTGA | | |
|  | egfp-r | AAAACCTAGGGCTCGTCCATGCCGAGAGTGATCCC | | |
| *ply187N* | ply187N-f | AAAACCATGGGGATGGCACTGCCTAAAACGGGT | | |
|  | ply187N-r | AAAAGGATCCTGGTGGTGTAGGTTTCGGTTCTGCT | | |
| *plyV12* | plyV12-f | AAAACCATGGGCATGAGTAACATTAACATGGAAAC | | |
|  | plyV12-r | AAAACTCGAGTTACTTAAATGTACCCCATGCTTCCT | | |
| *V12C* | V12C-f | GACGGAATTCTTAAACGGTGGAAGCACTCCTCCAAAAC | | |
|  | V12C-r | TCGCCTCGAGTTACTTAAATGTACCCCATGCTTCCTTACC | | |

**Fig. S1 Inhibition zone of *S. aureus* strains in the plate lysis assay. *S. aureus* M1, 391and 2080 are isolated from milk produced by cow with mastitis.**


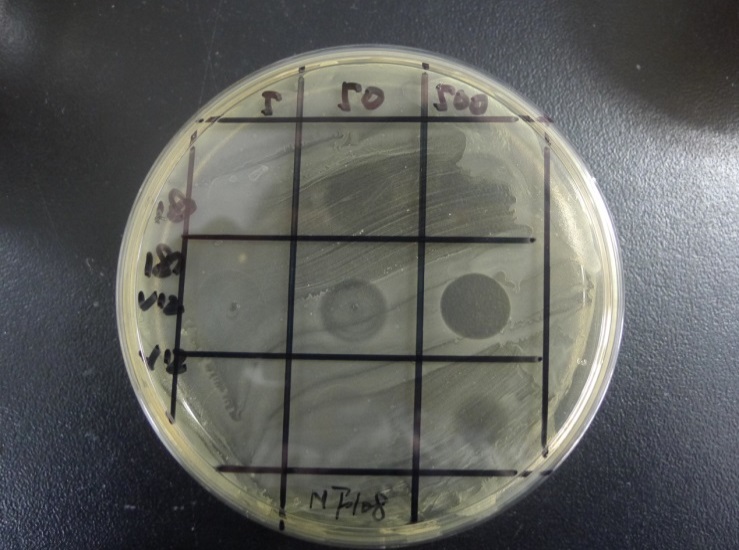

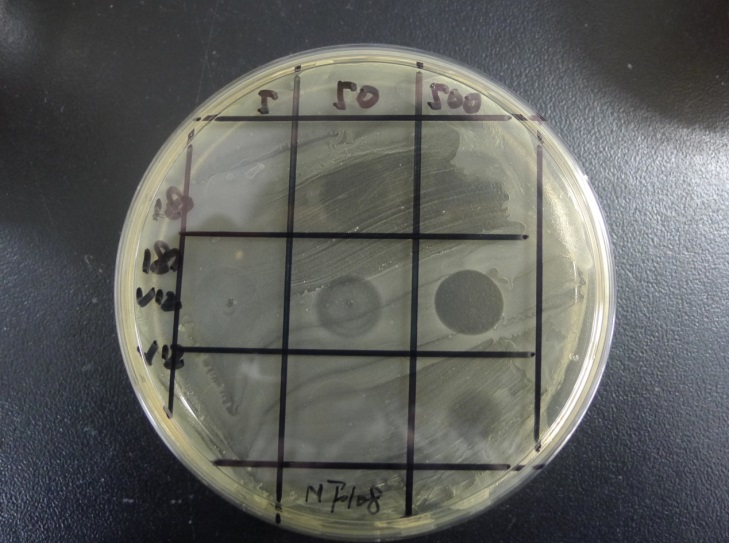

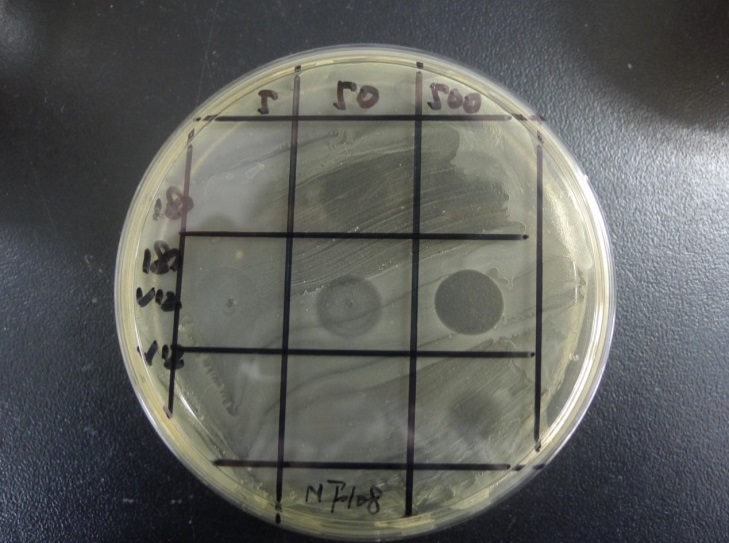


AM001


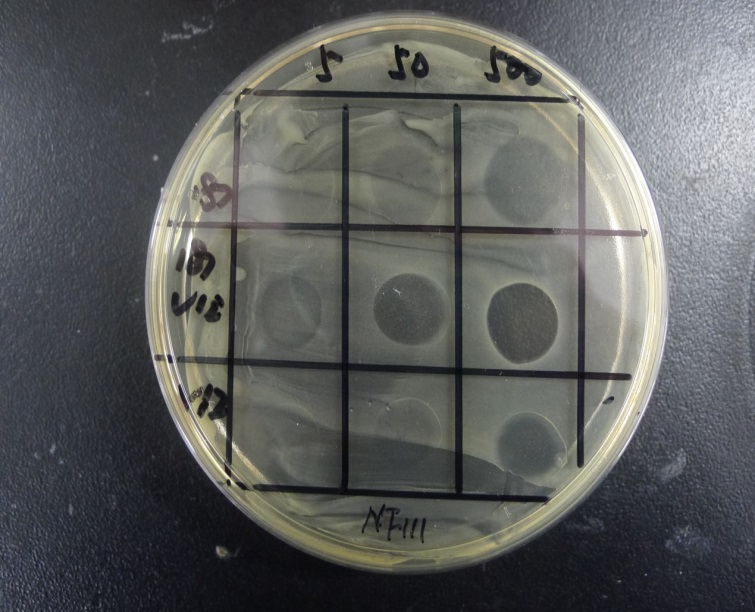

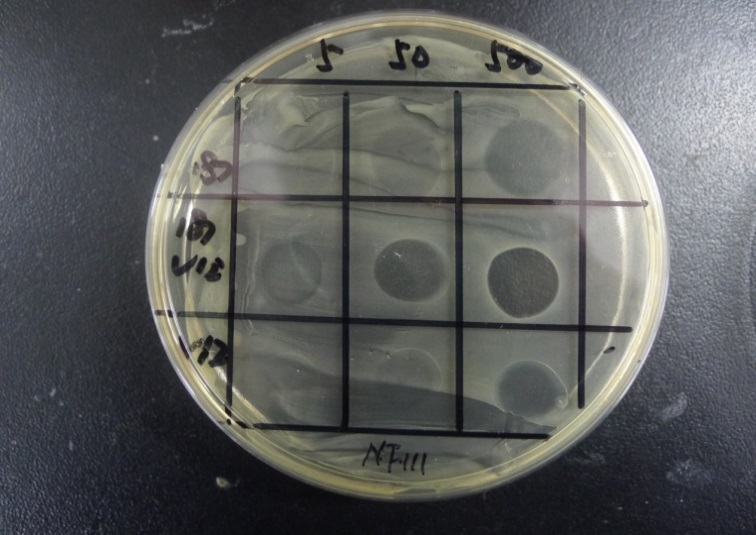

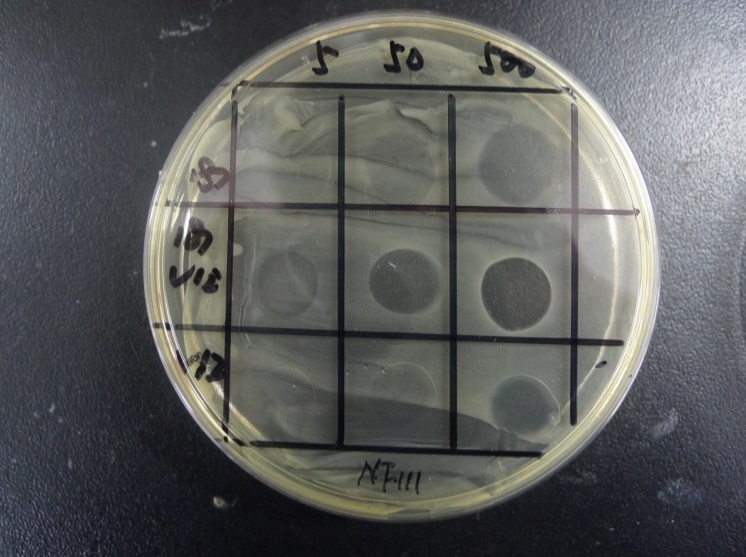


AM002


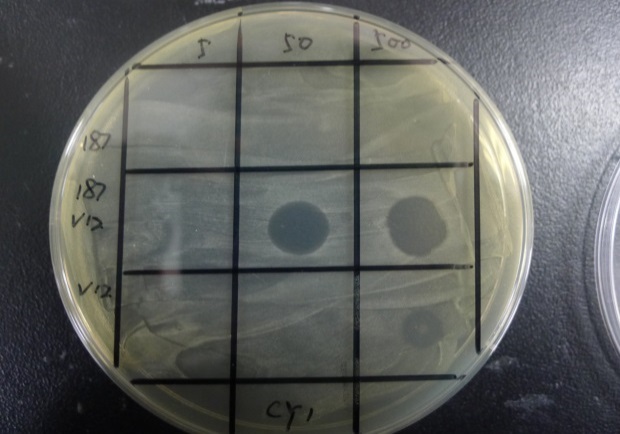

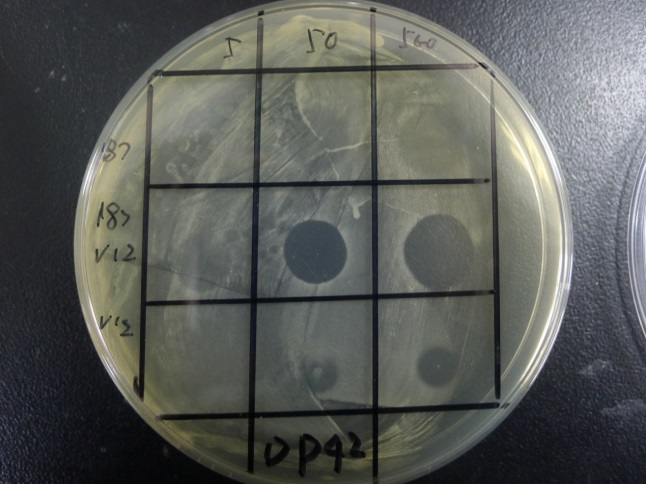

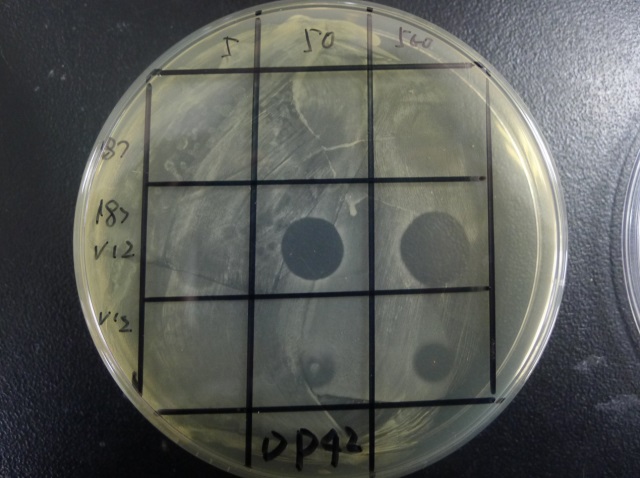


AM005


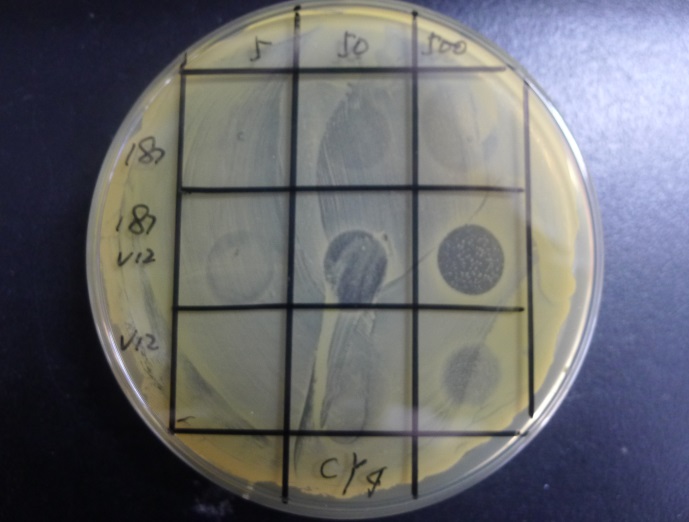

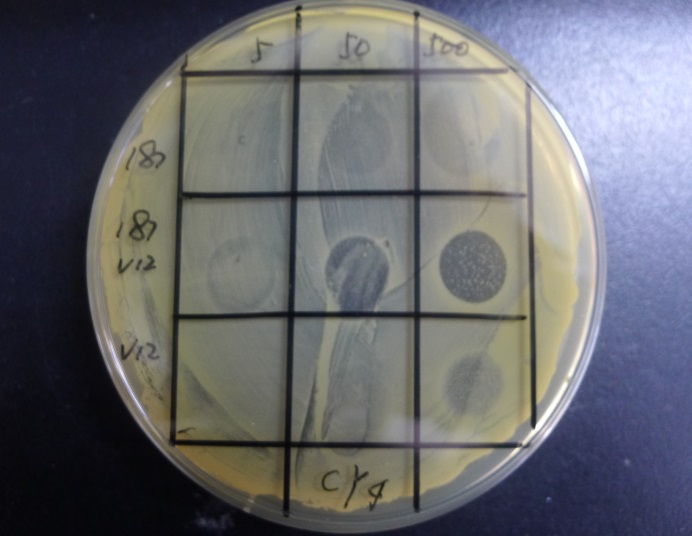

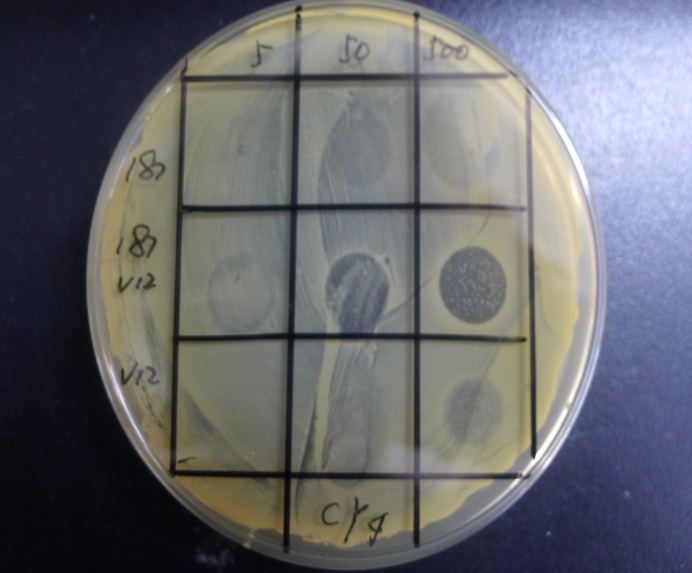


AM006


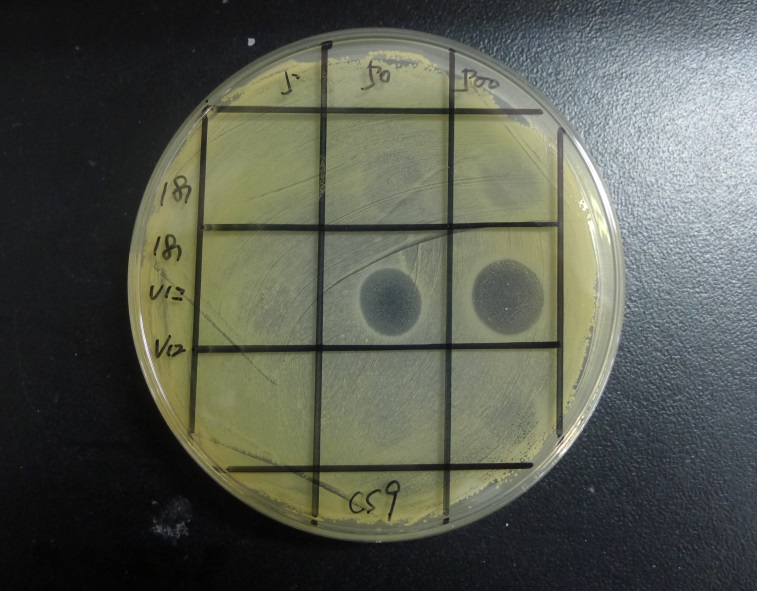

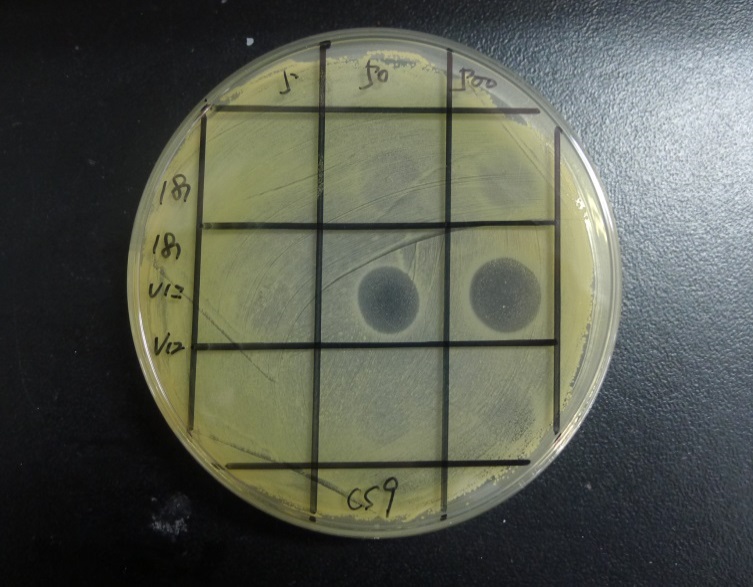

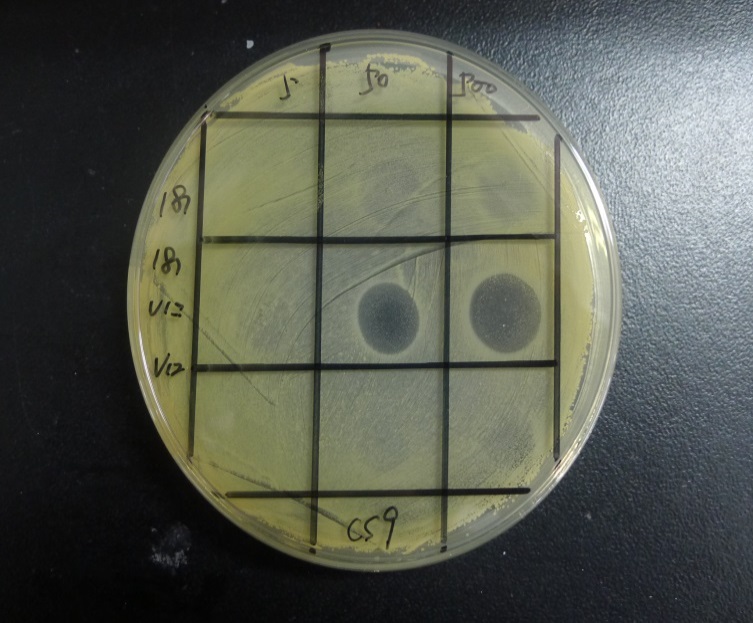


AM008


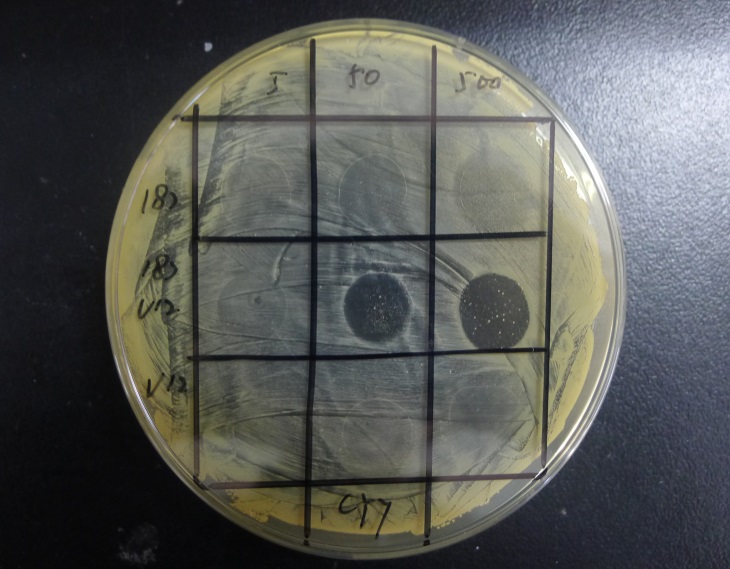

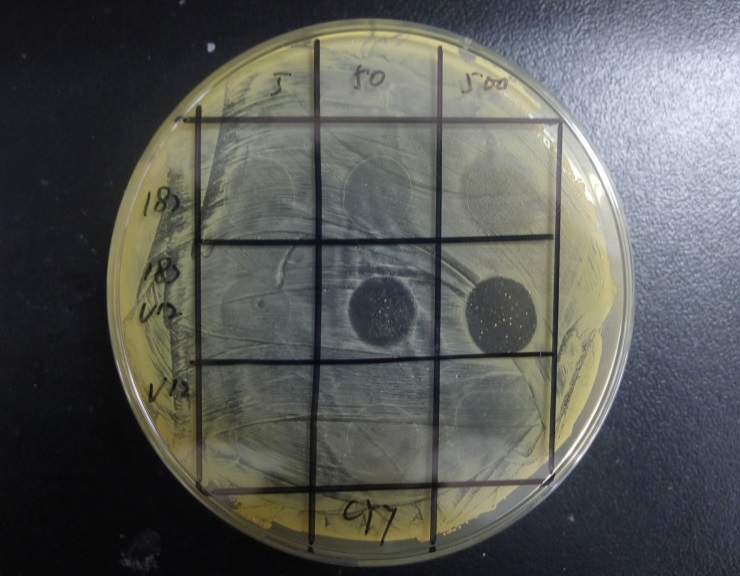

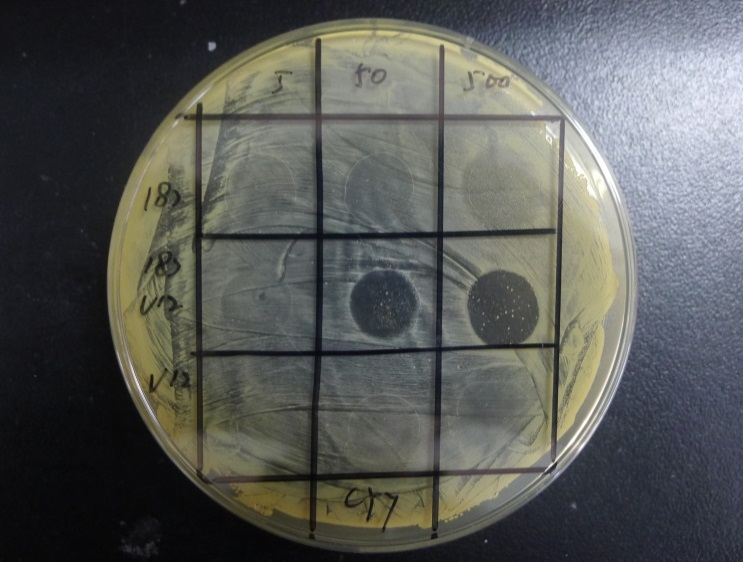


AM010


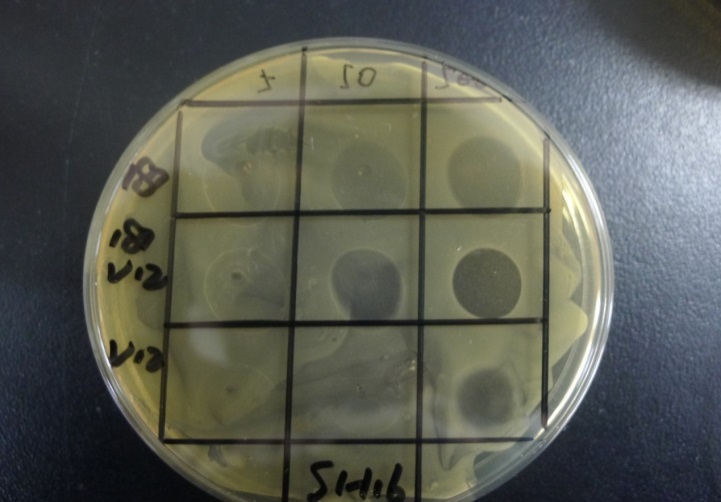

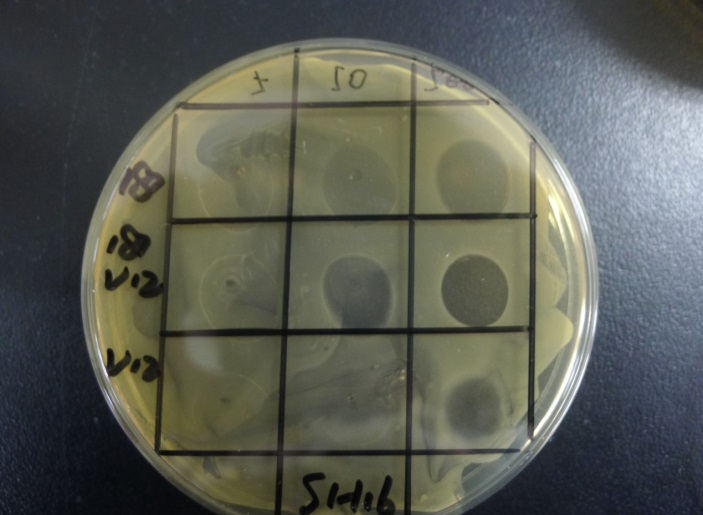

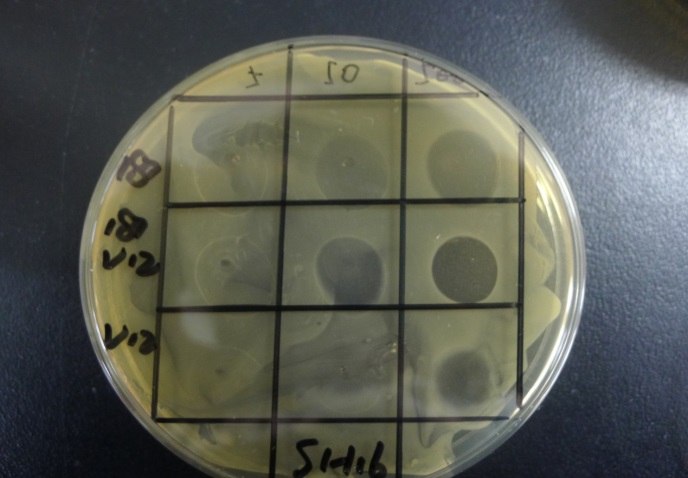


AM014


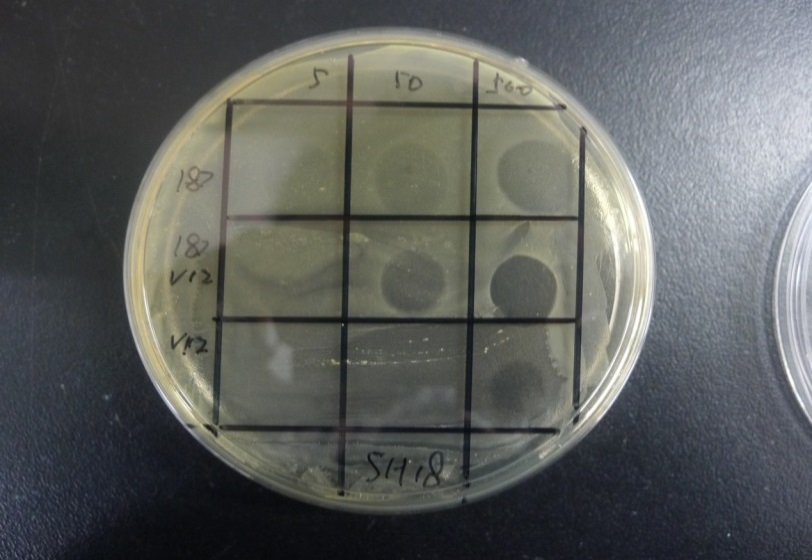

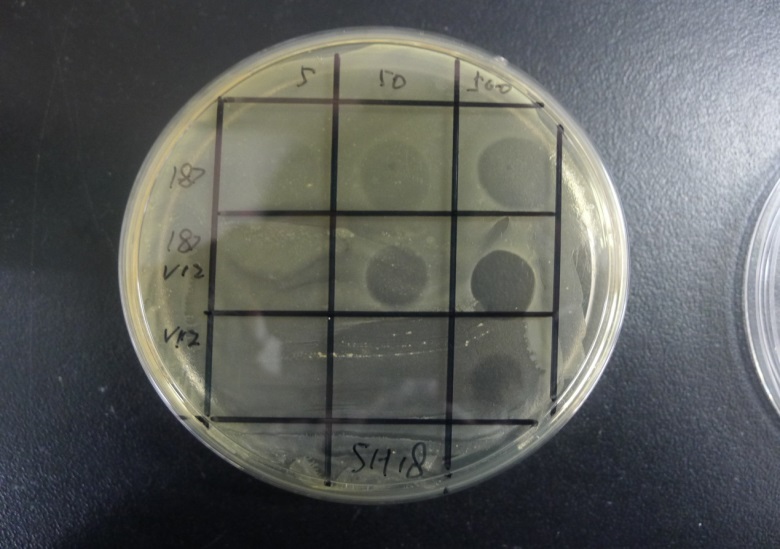

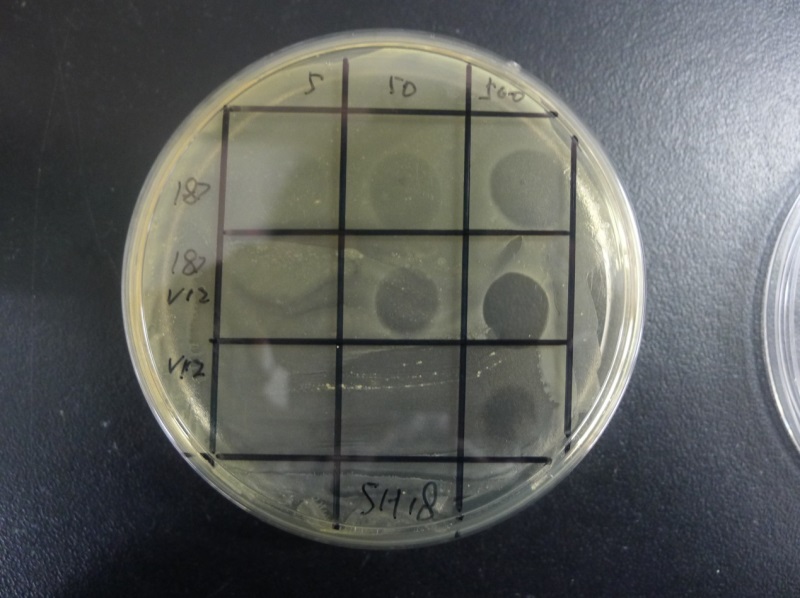


AM016


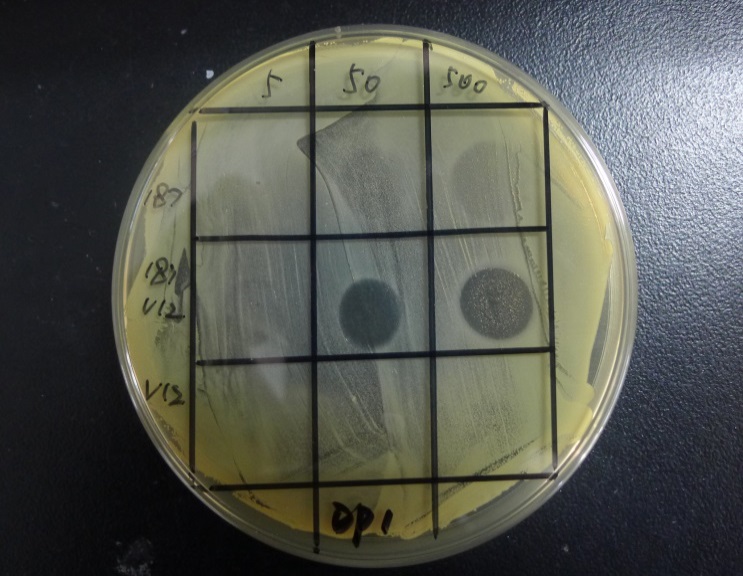

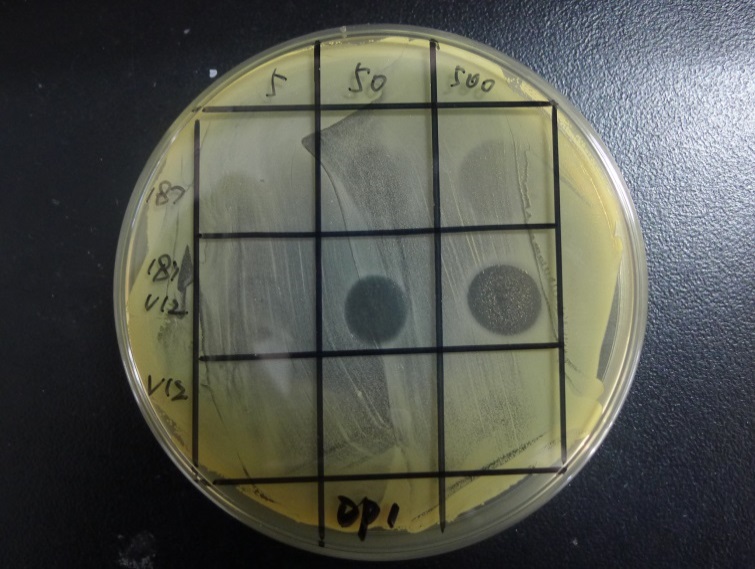

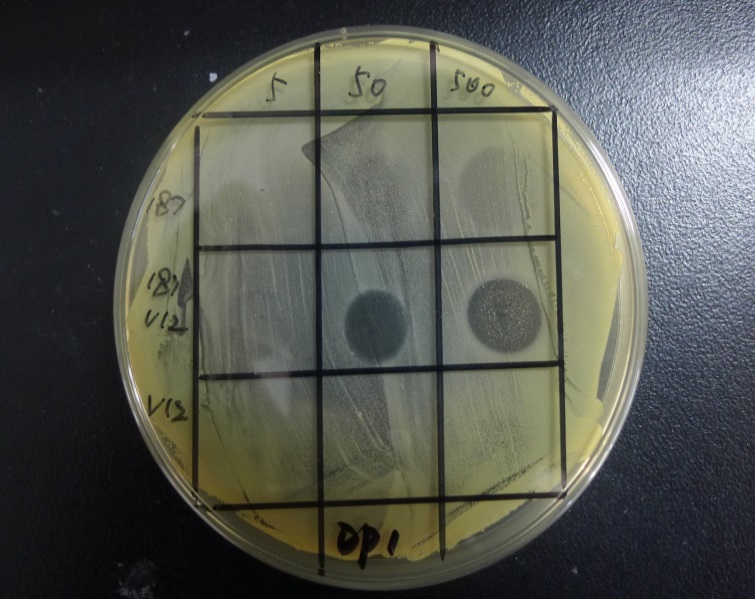


AM027


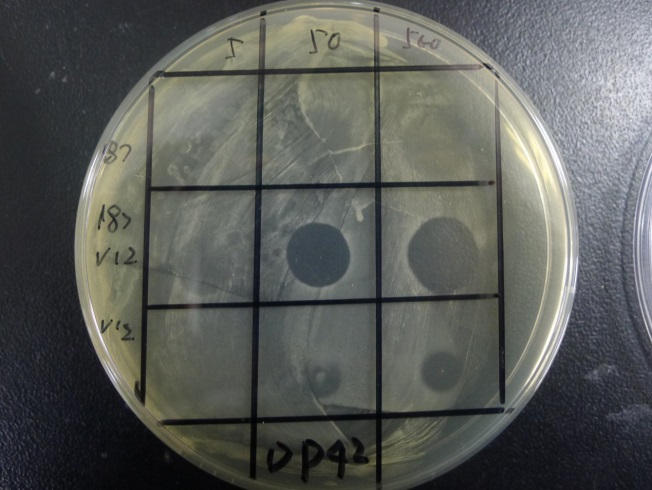

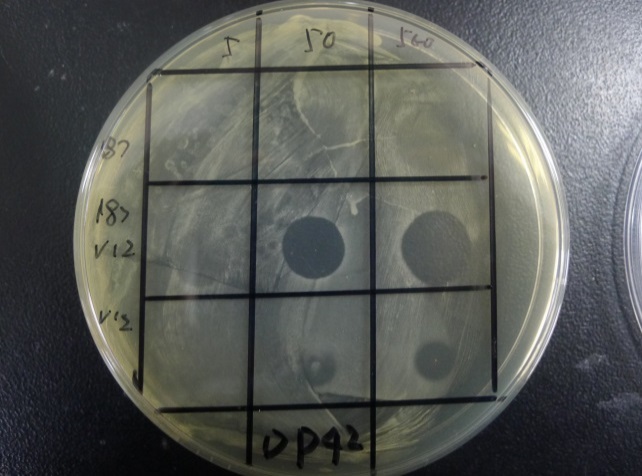

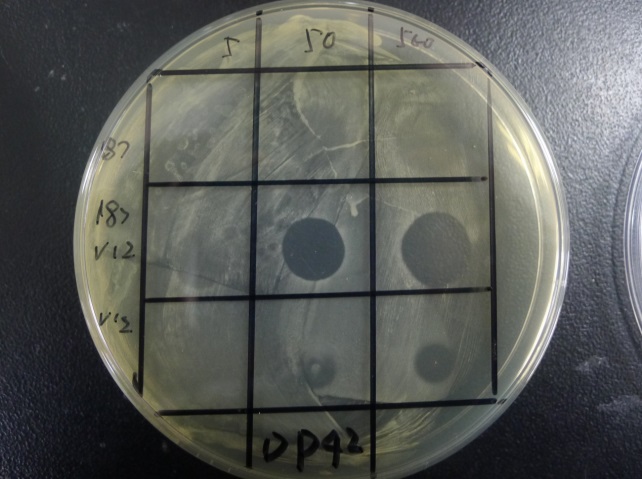


AM031


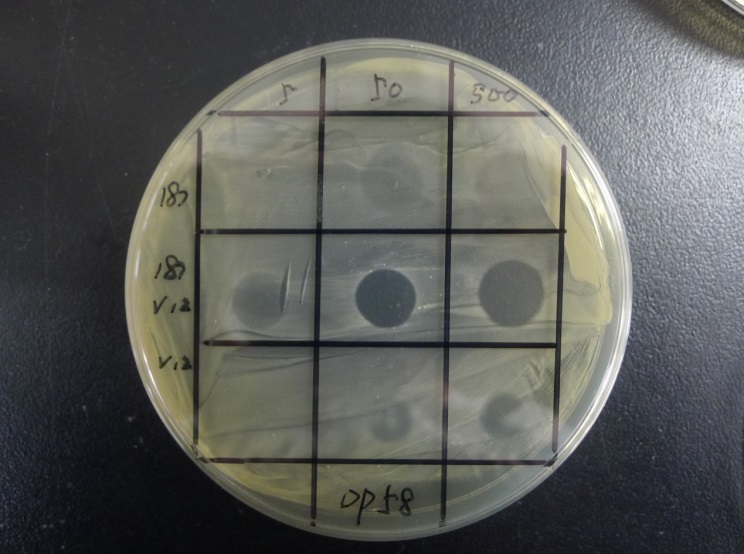

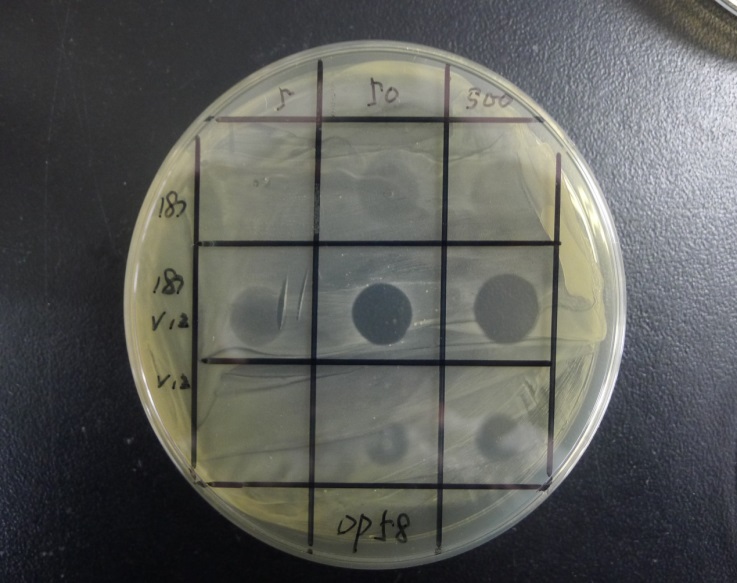

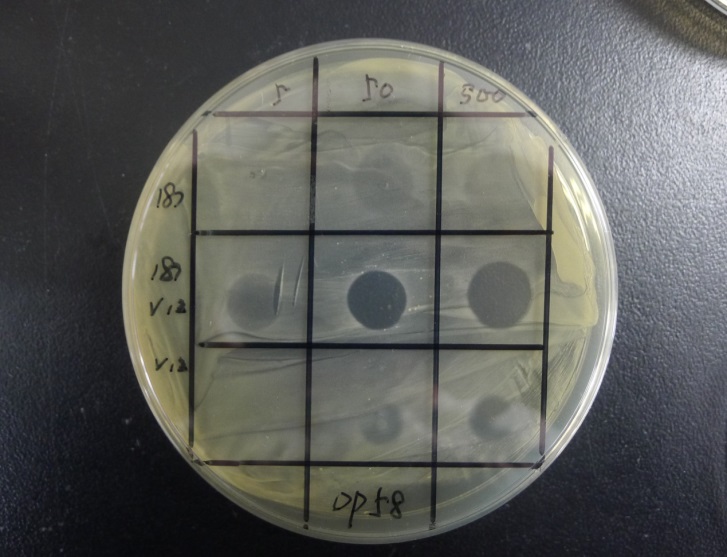


AM032


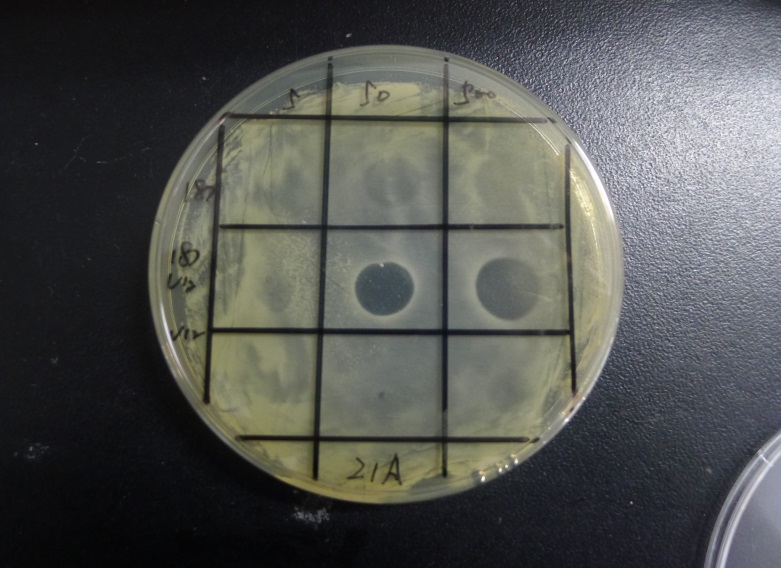

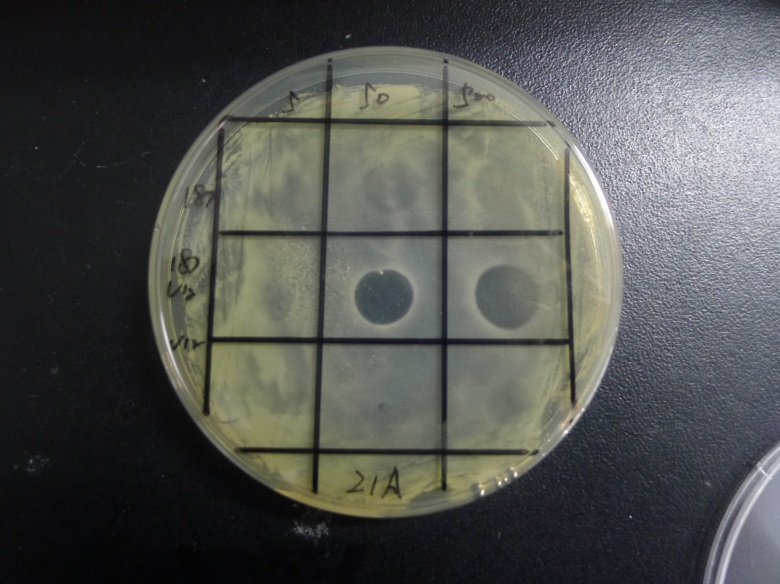

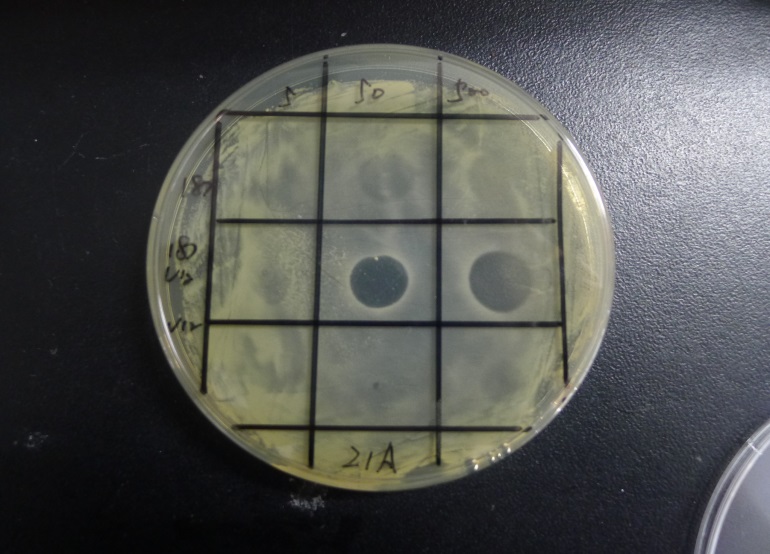


AM037


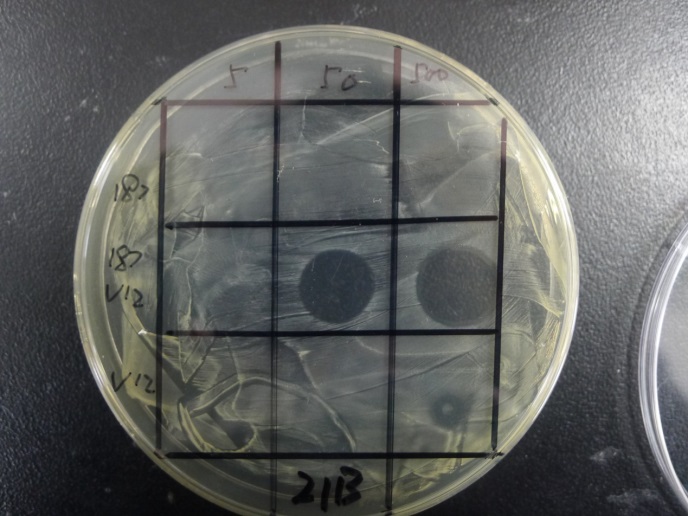

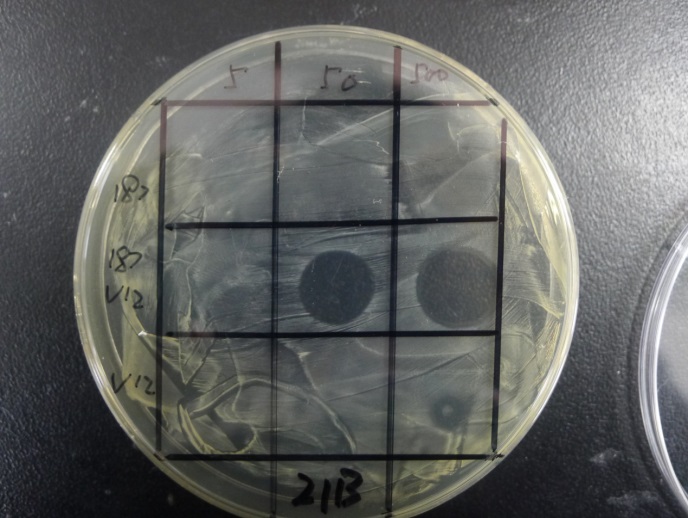


AM038


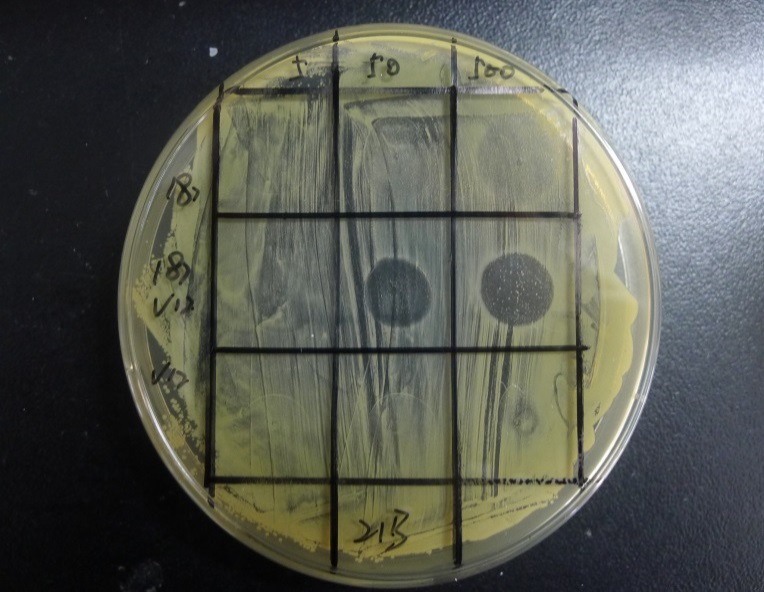

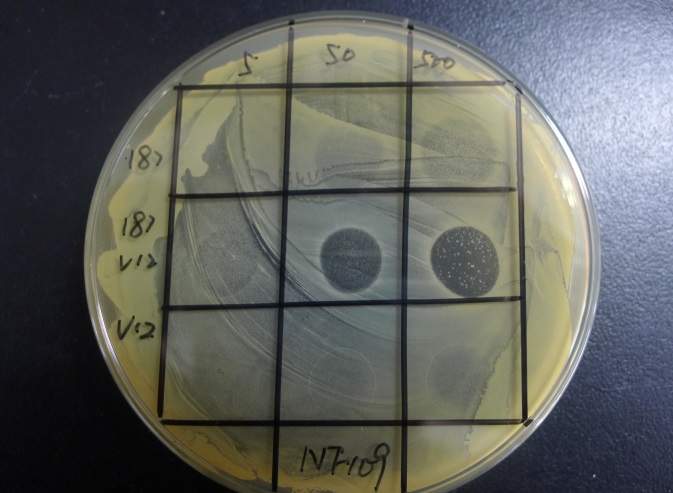

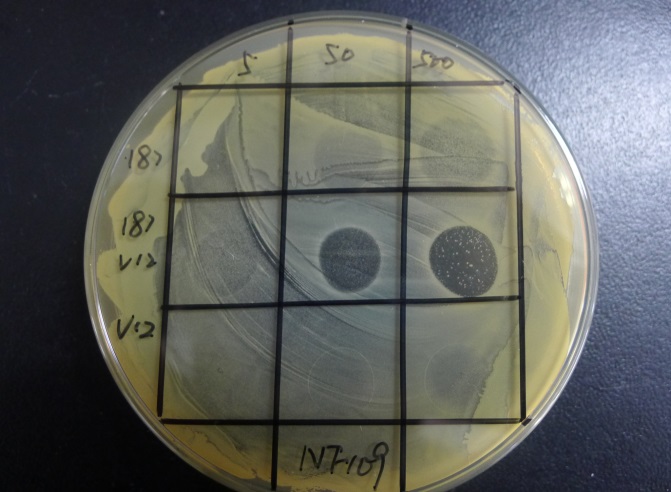

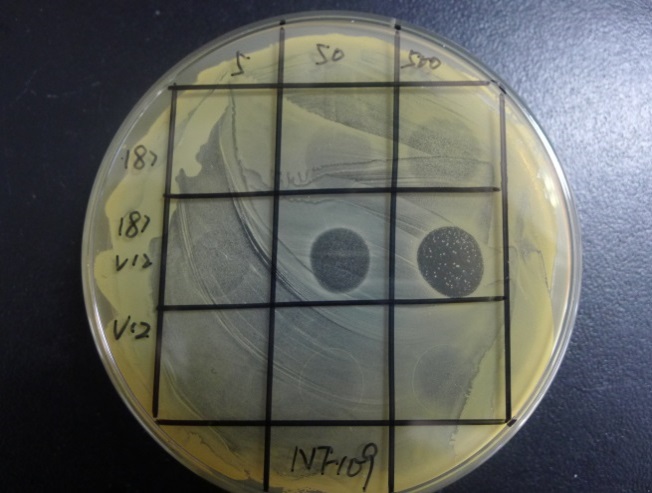


AM043

Ply187N

PlyV12

Ply187N-V12C

5 pmol

50 pmol

500 pmol

5 pmol

50 pmol

500 pmol

5 pmol

50 pmol

500 pmol


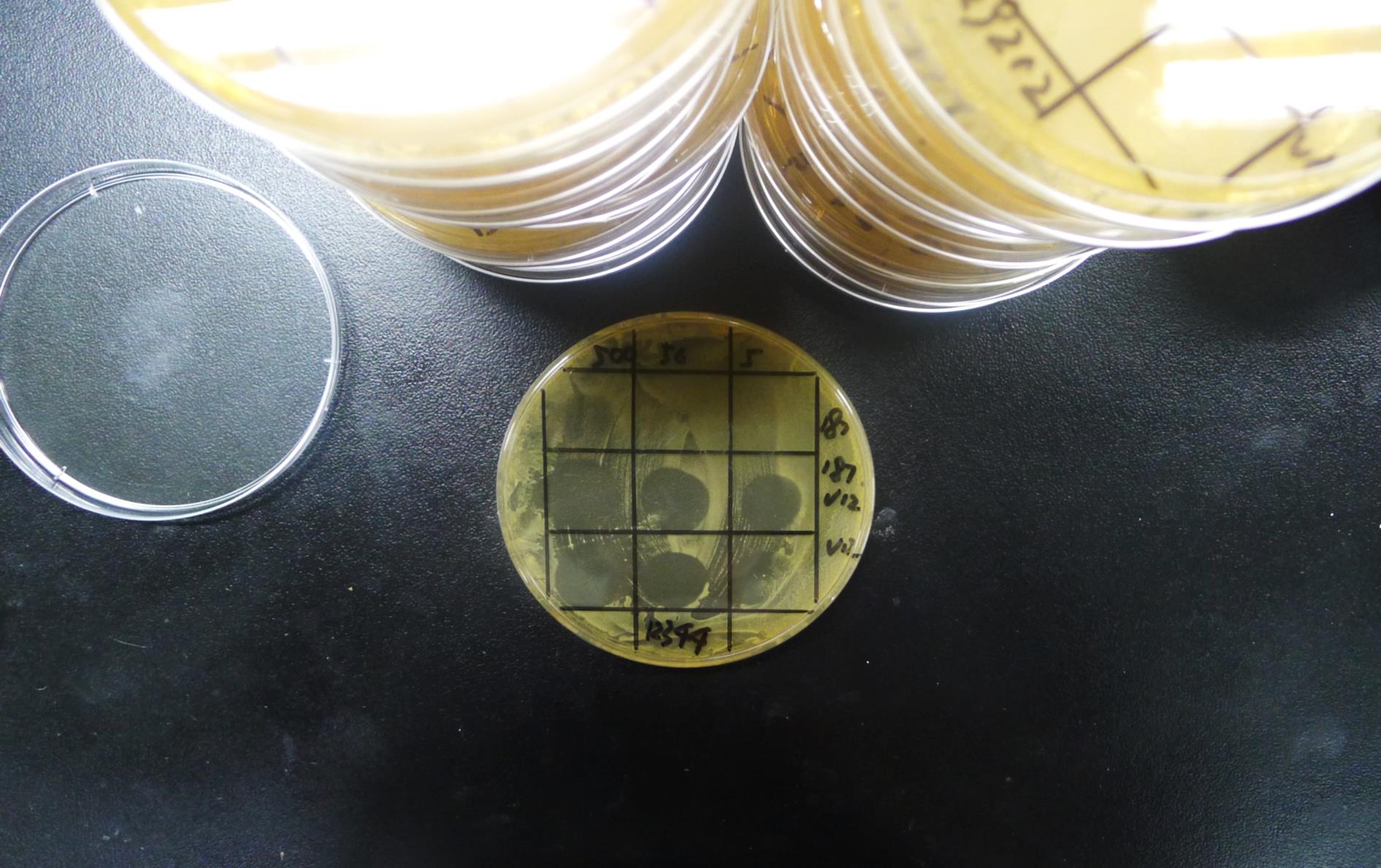

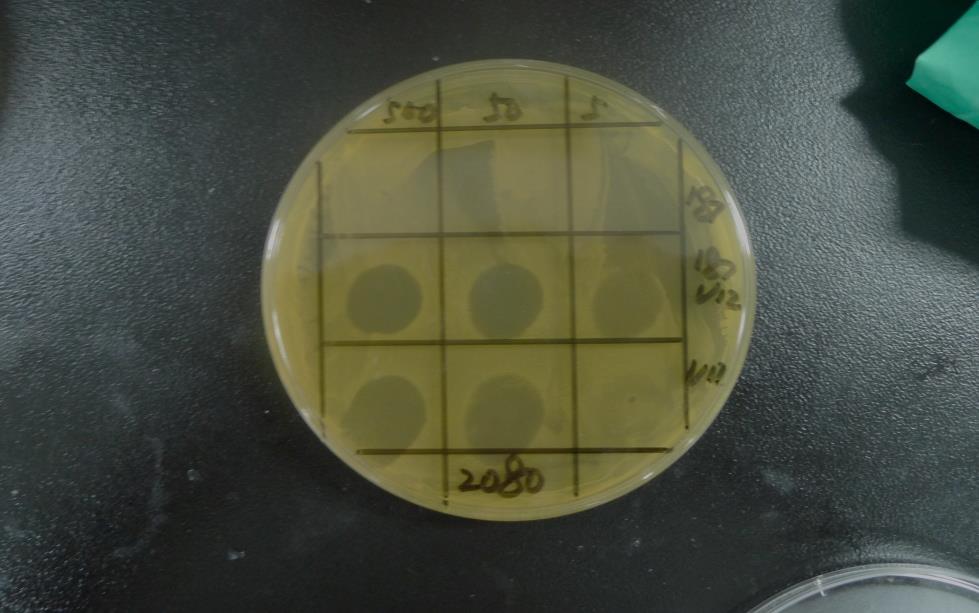

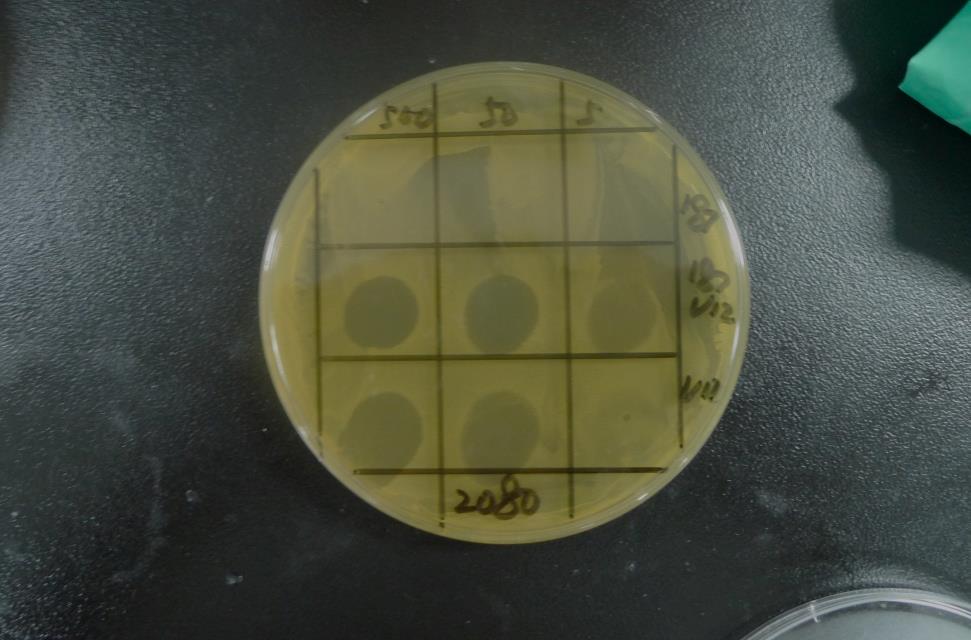


M1


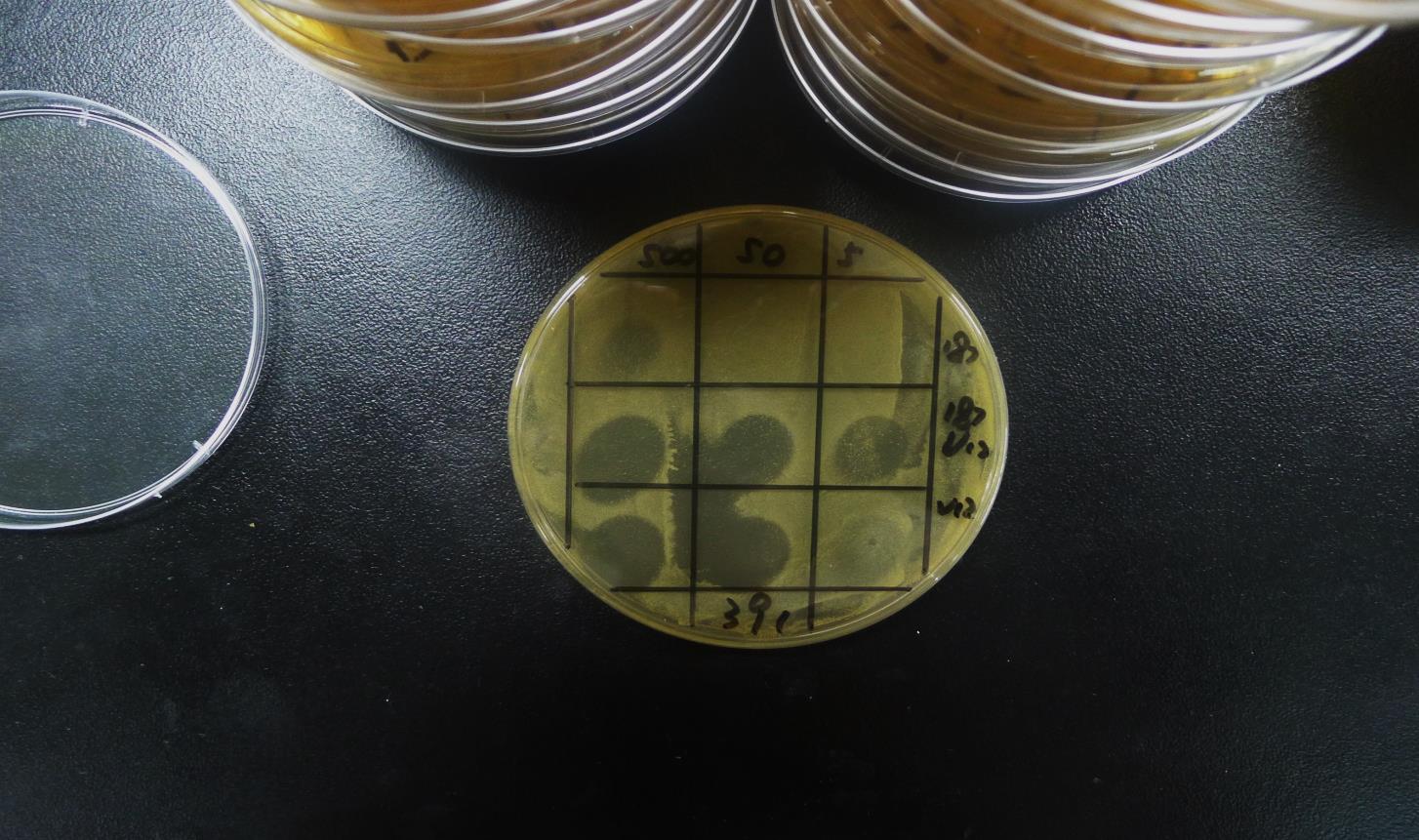

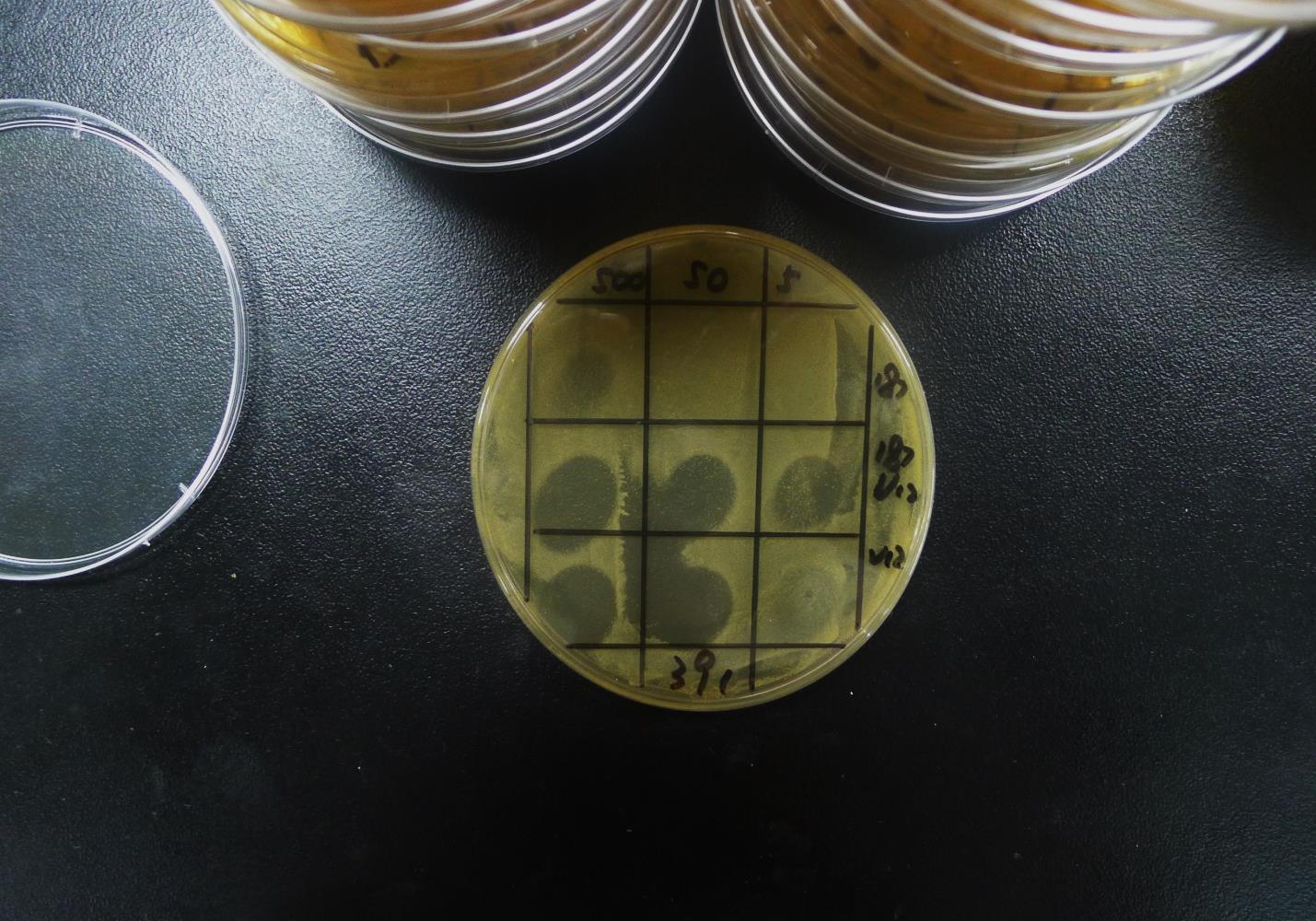

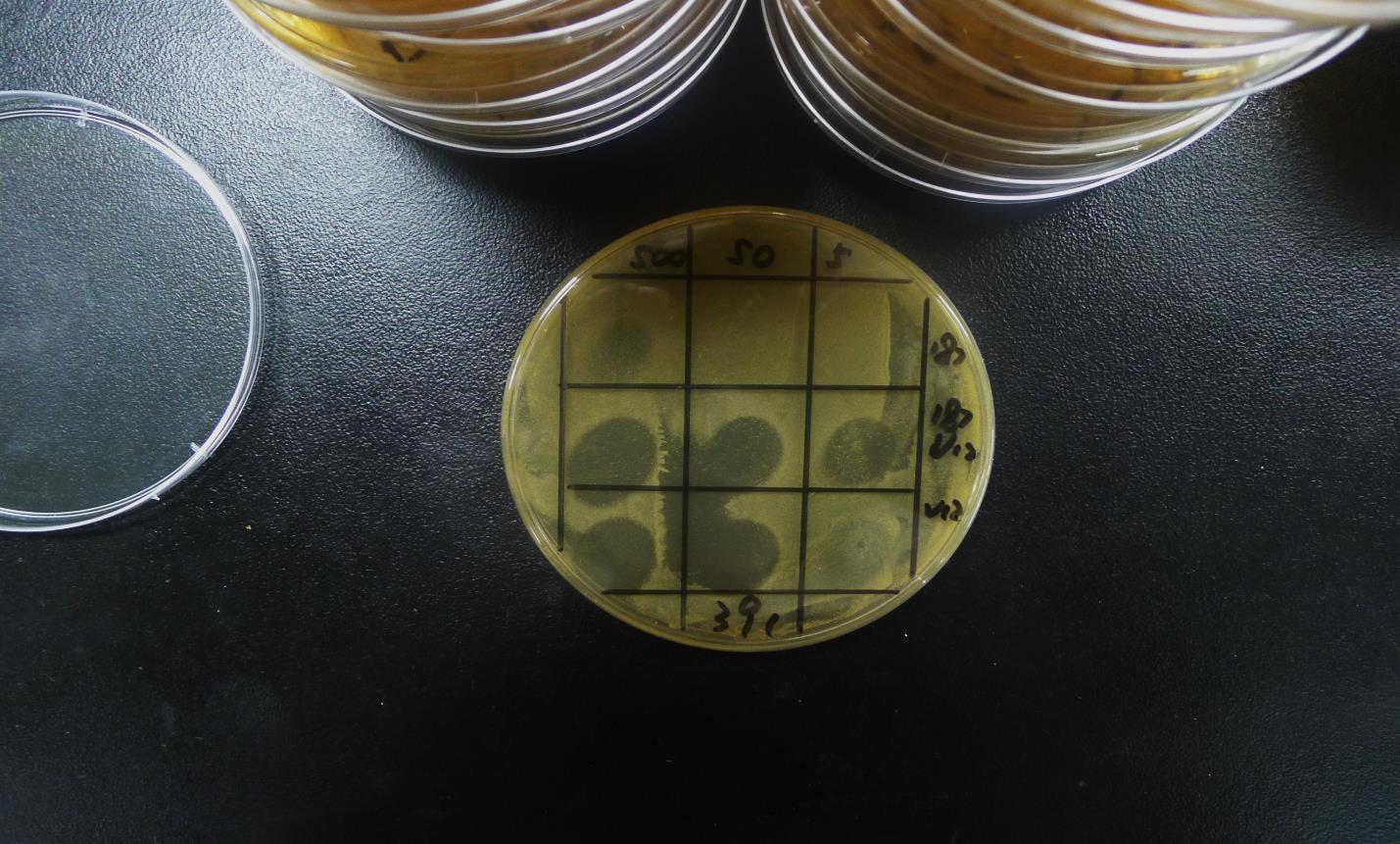


391


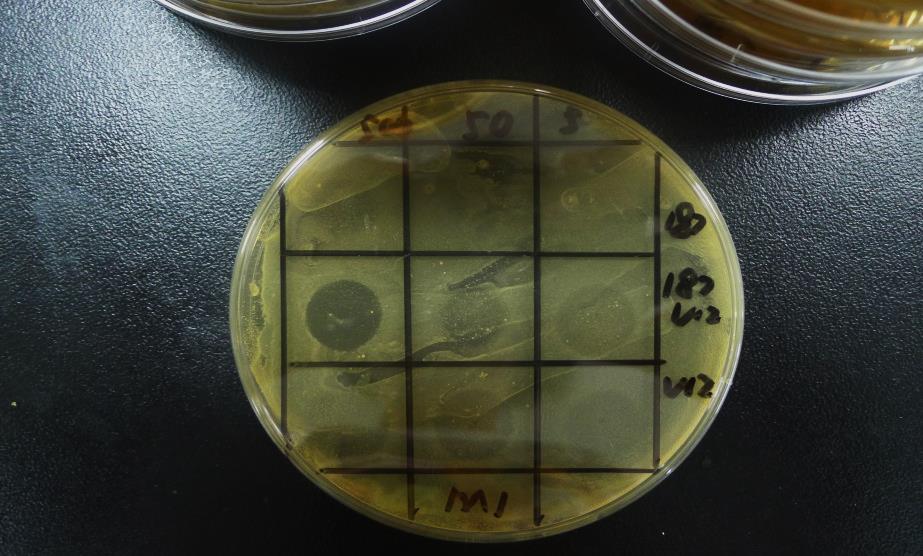

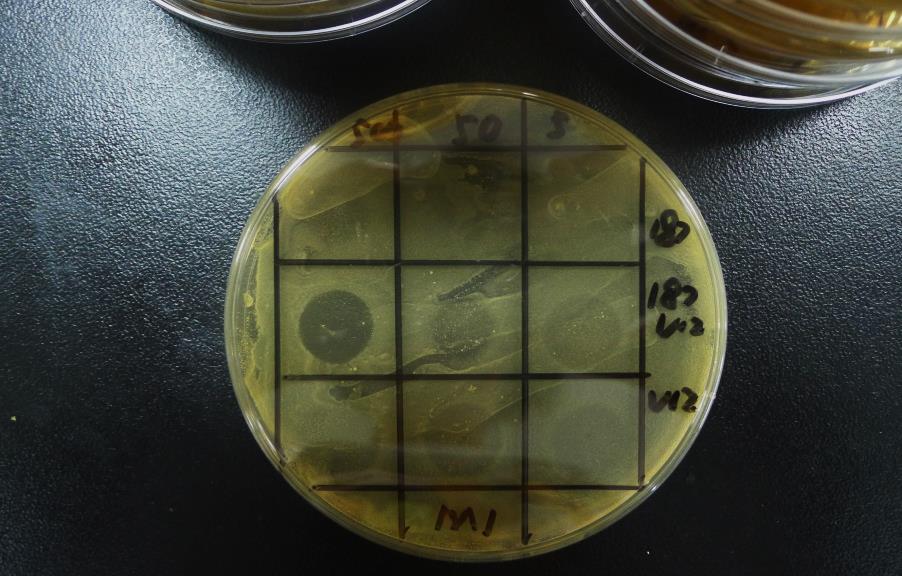

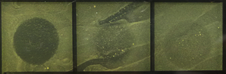


2080


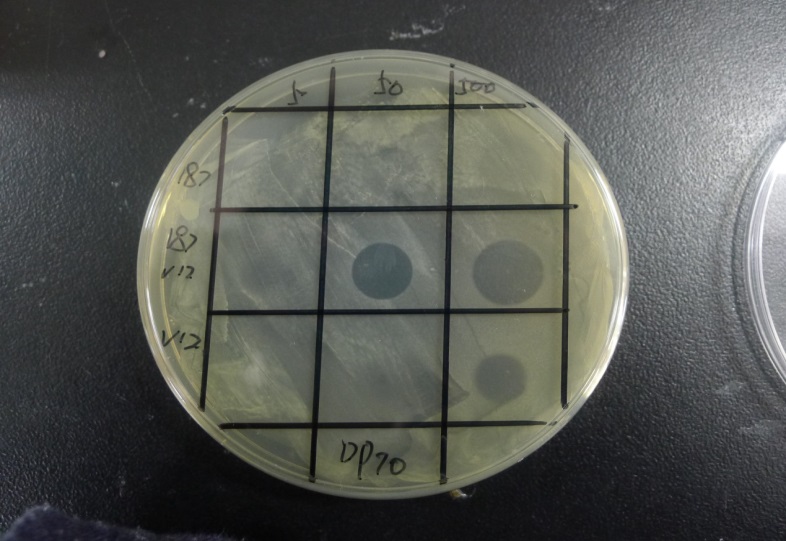

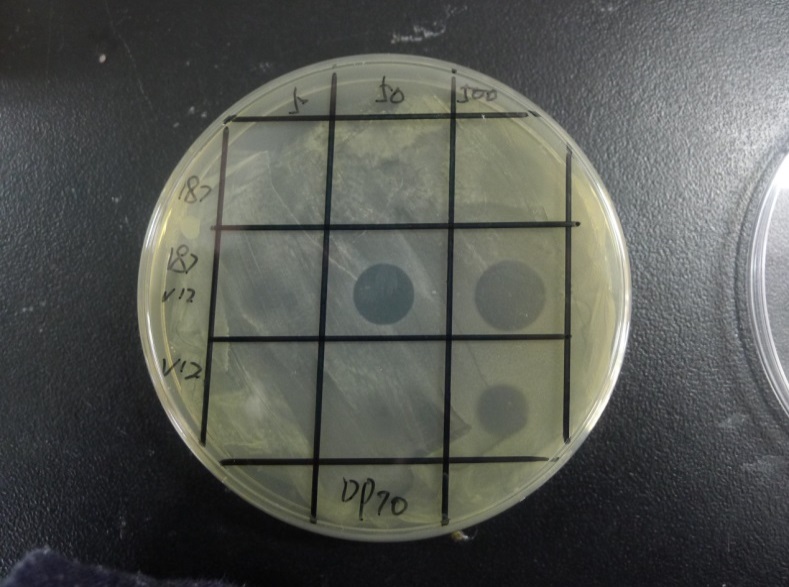

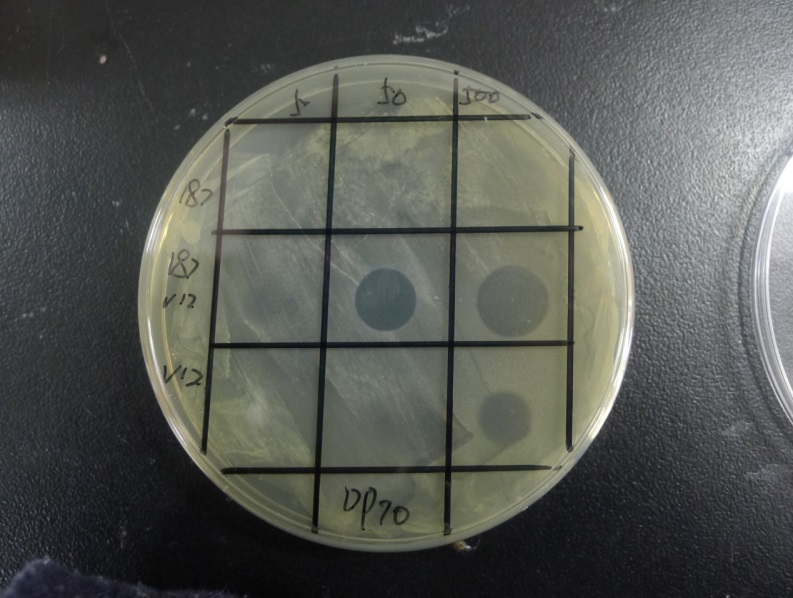


AM045


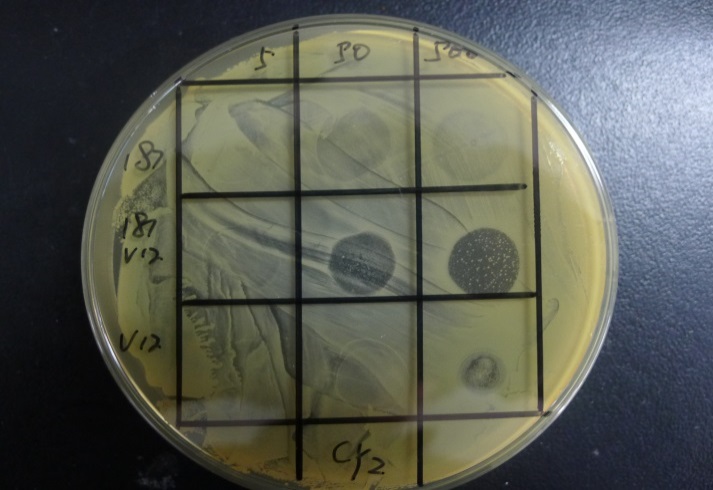

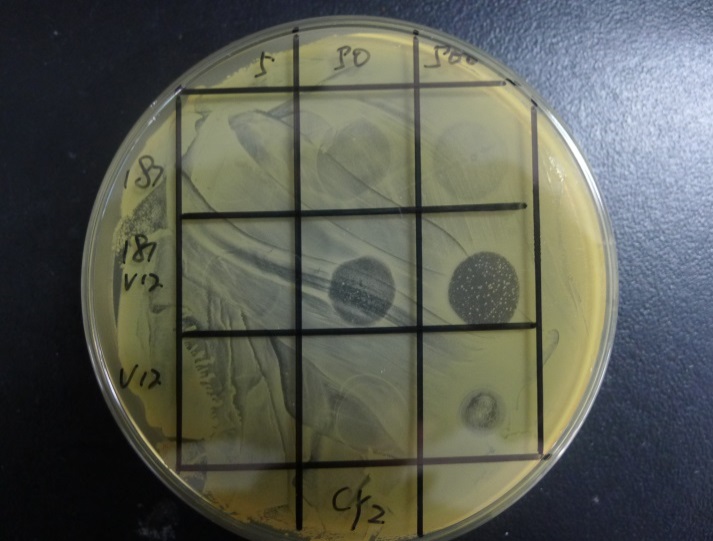

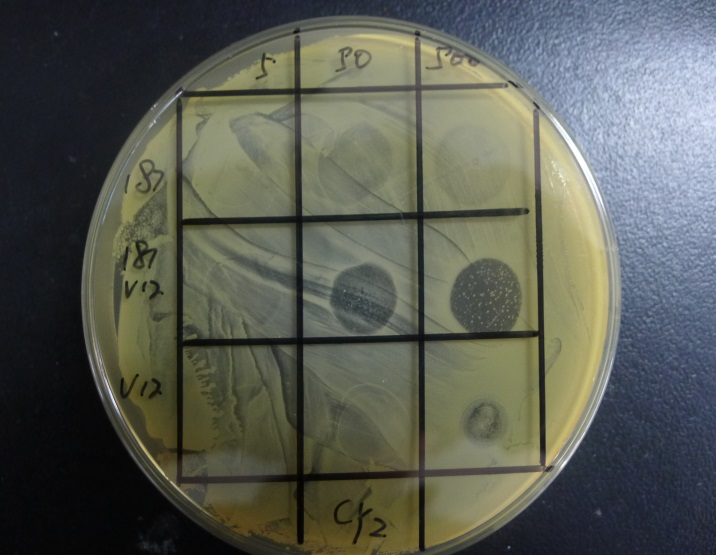


AM046


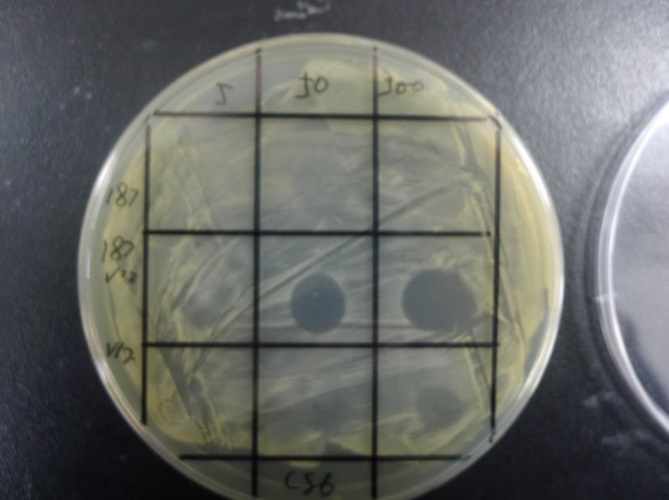

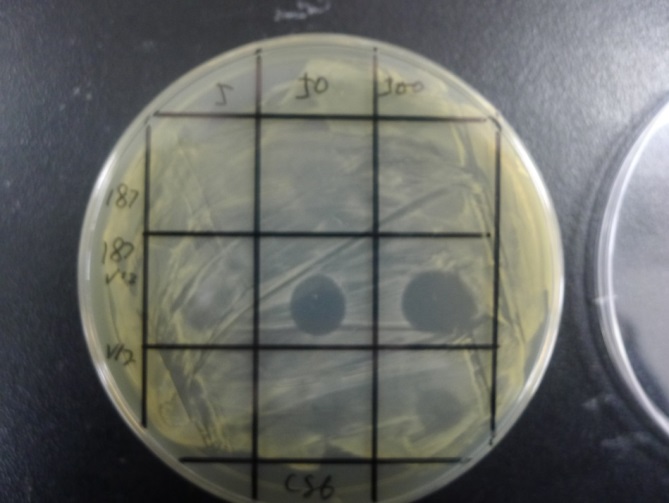

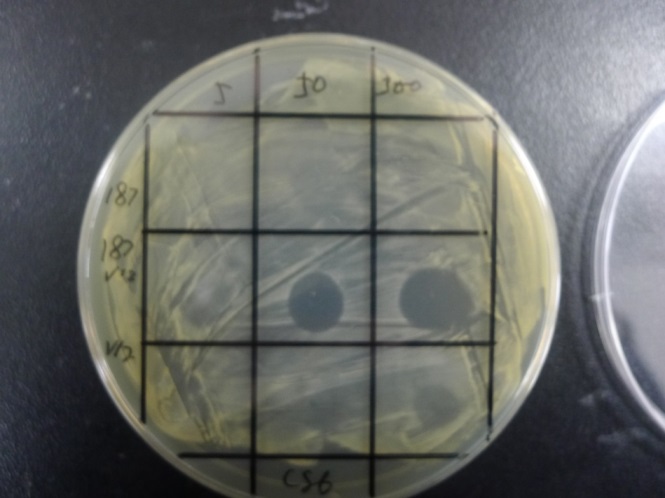


AM048


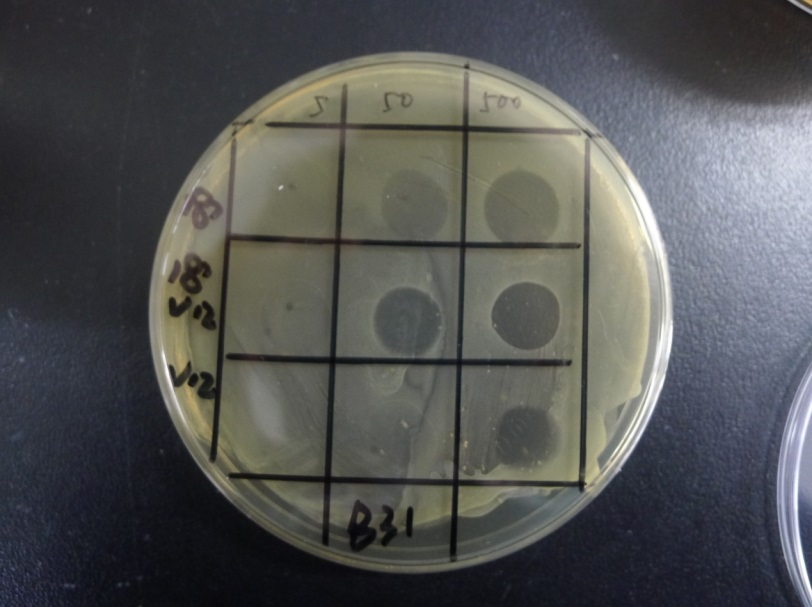

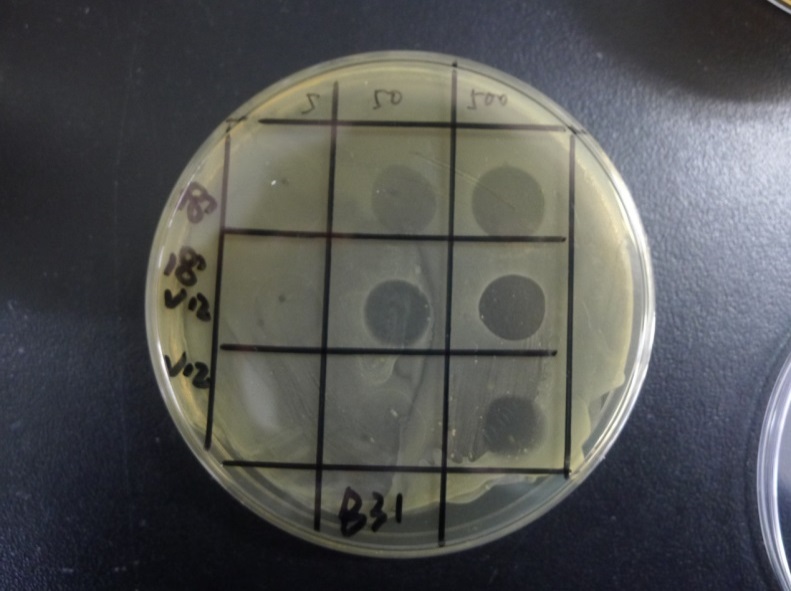

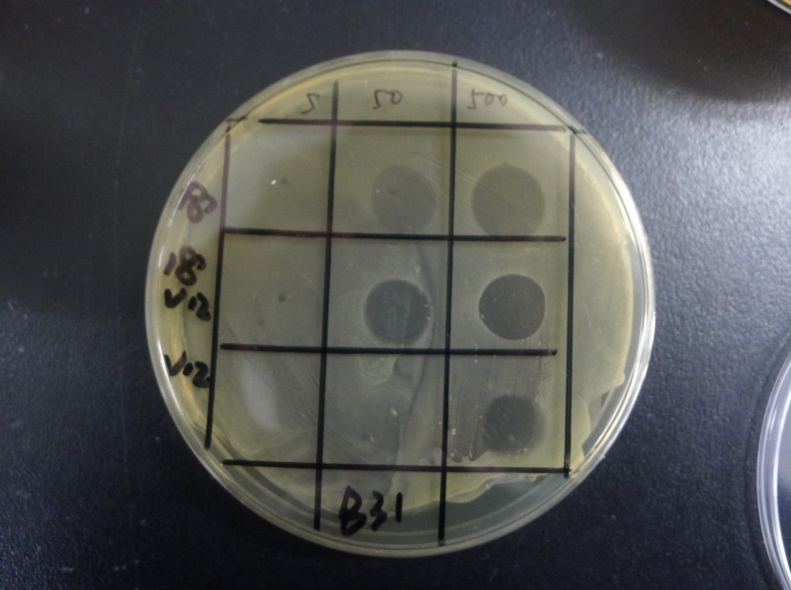


AM054


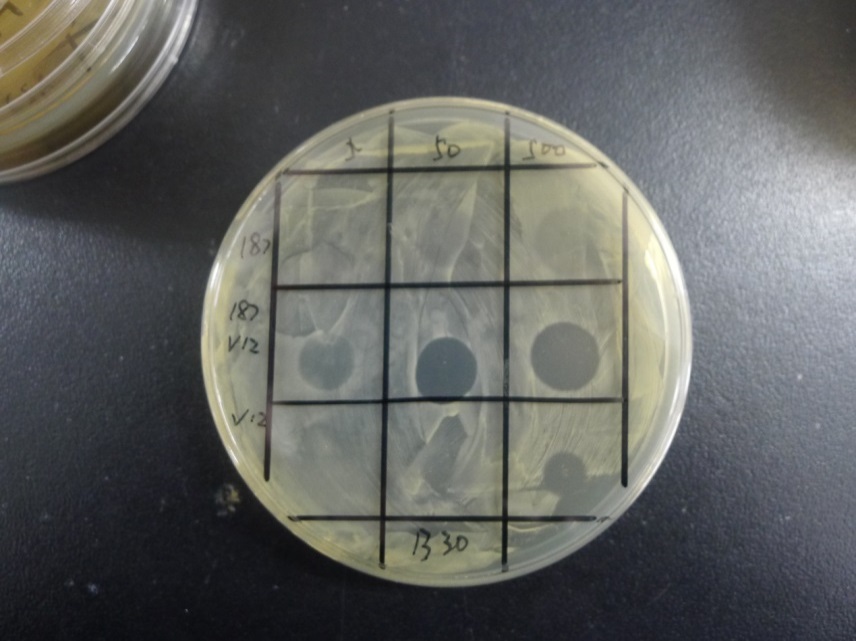

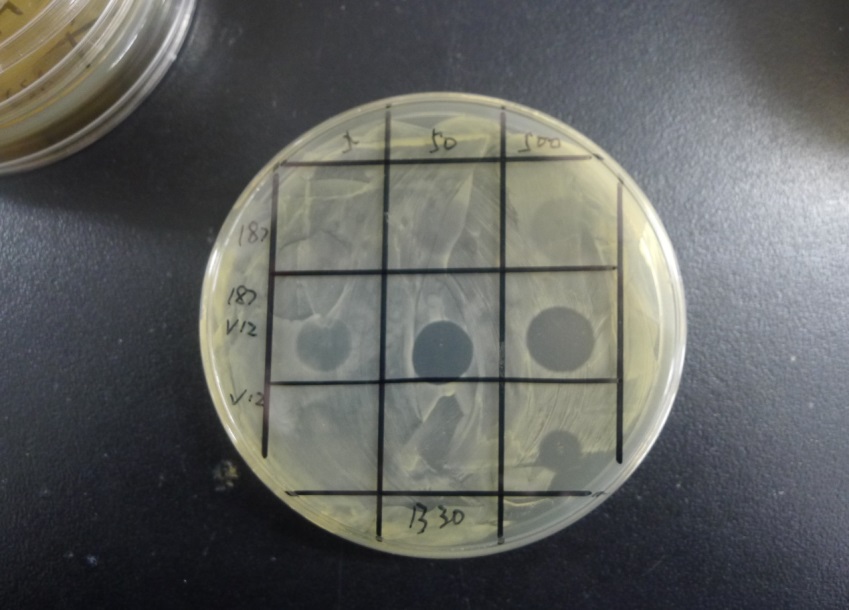

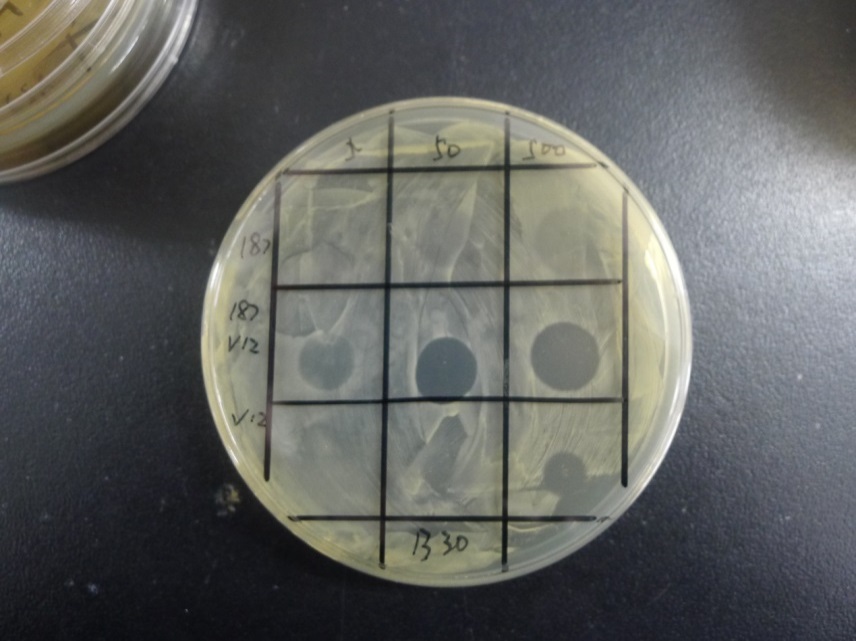


AM058


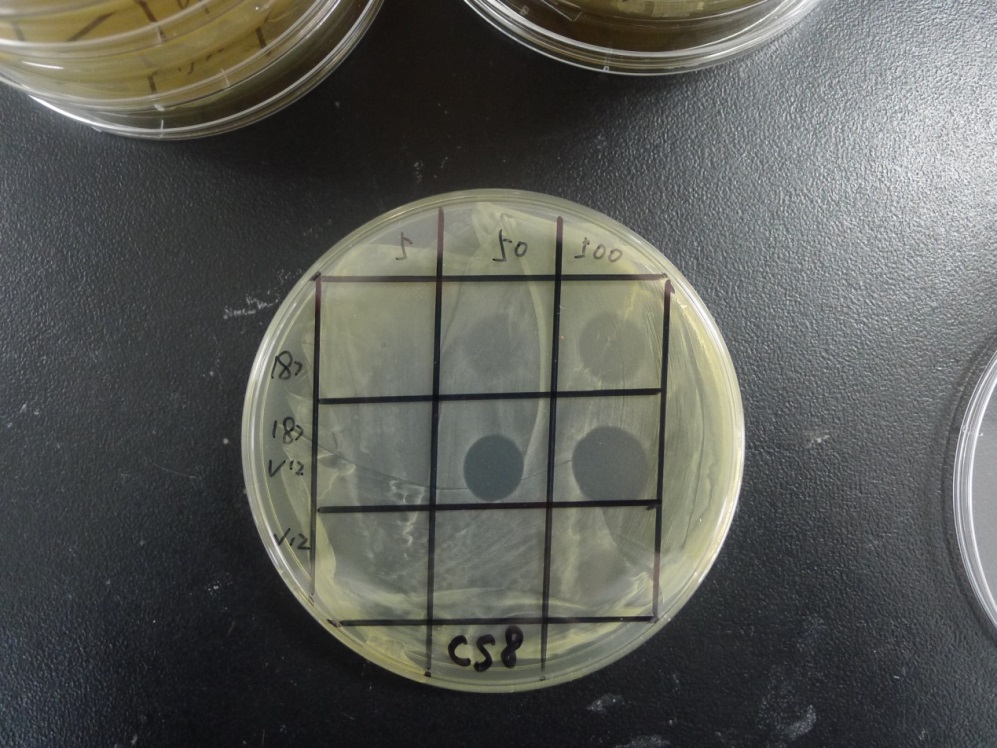

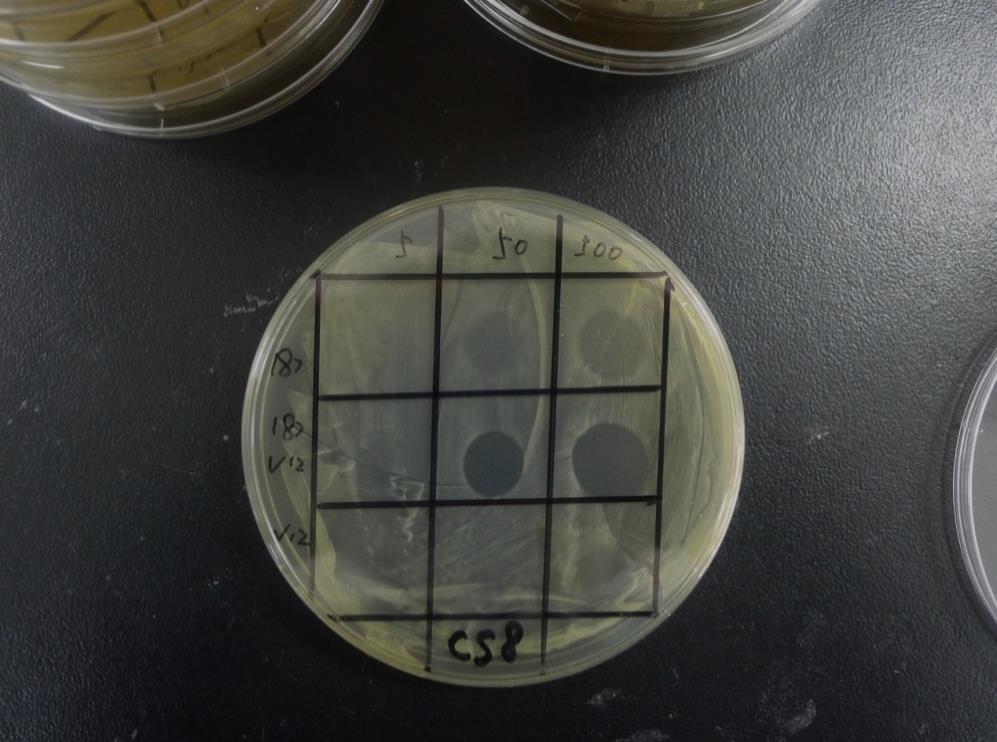

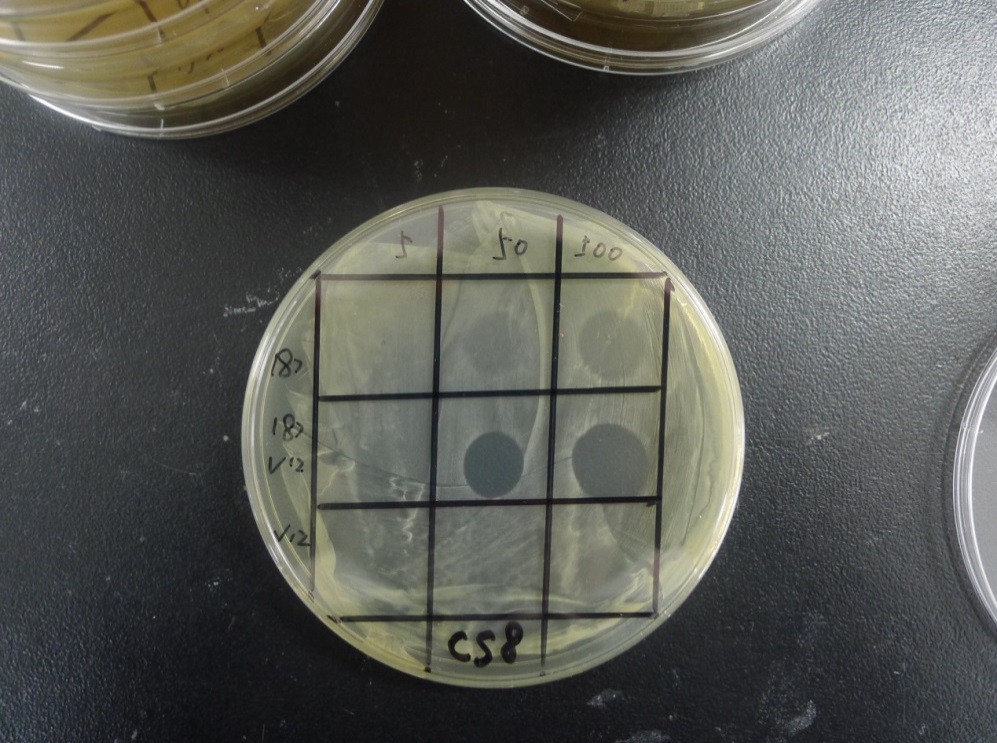


AM061


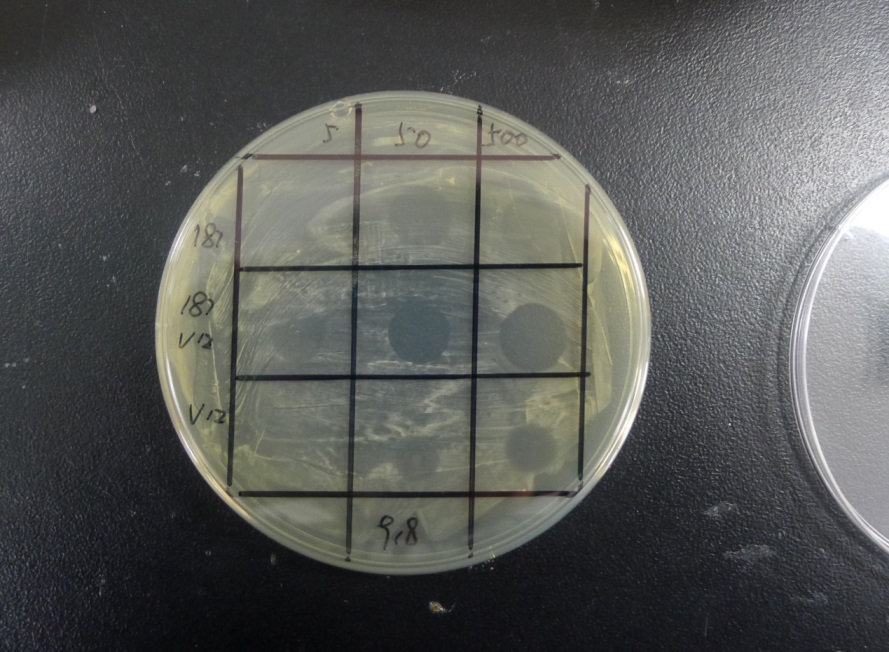

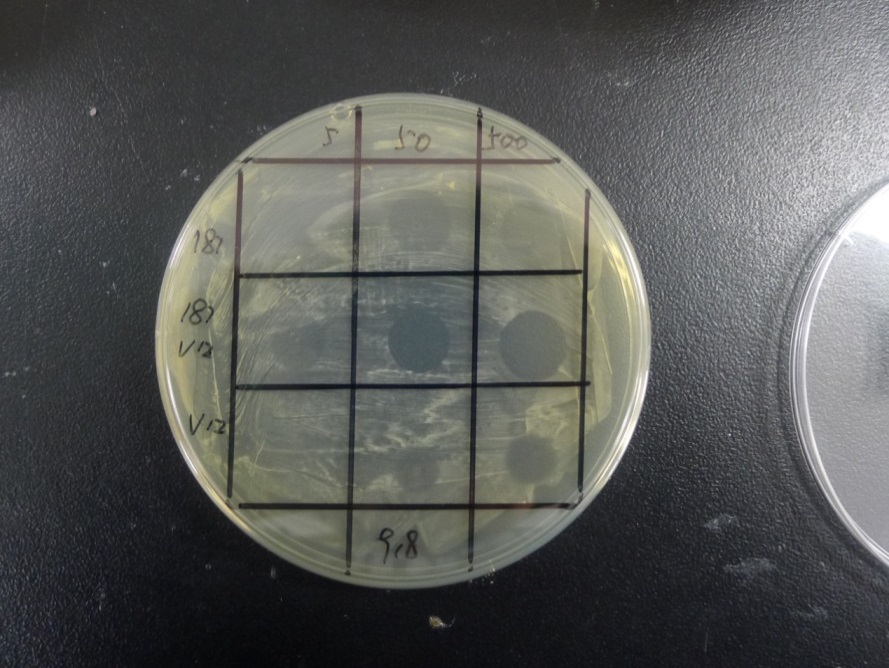

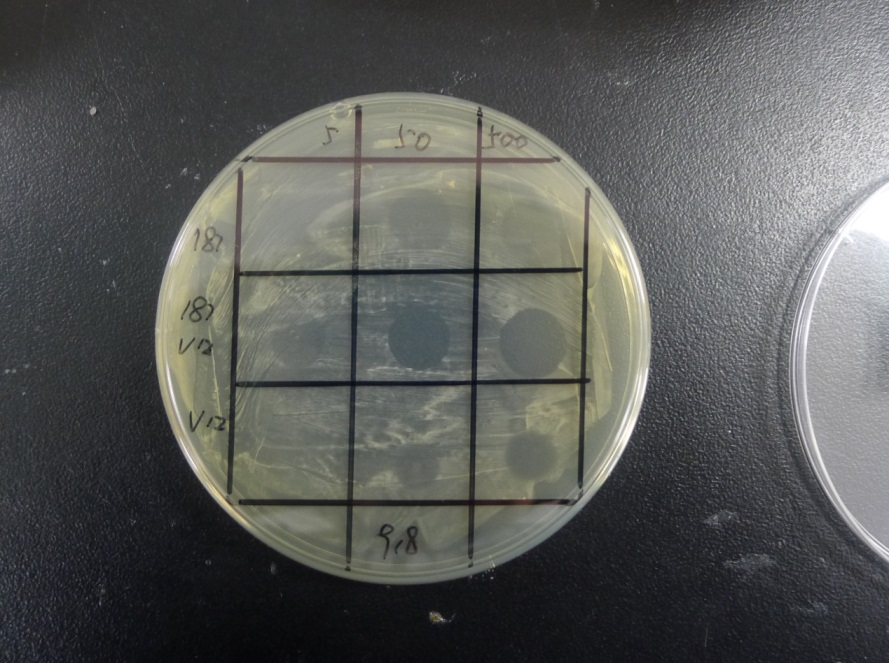


AB9118
